# The *SITS framework*: sustaining innovations in tertiary settings

**DOI:** 10.3389/frhs.2023.1102428

**Published:** 2023-06-08

**Authors:** Letitia Nadalin Penno, Ian D. Graham, Chantal Backman, Barbara Davies, Janet Squires

**Affiliations:** ^1^ Faculty of Environment and Health Sciences, Canadore College, North Bay, ON, Canada; ^2^ School of Nursing, Faculty of Health Sciences, University of Ottawa, Ottawa, ON, Canada; ^3^ School of Epidemiology and Public Health, University of Ottawa, Ottawa, ON, Canada; ^4^ Centre for Implementation Research, Ottawa Health Research Institute, Ottawa, ON, Canada

**Keywords:** frameworks/models/theories, sustainability, evidence-based practices, guidelines, interventions, innovations, adherence, nursing

## Abstract

**Background:**

To date, little attention has focused on what the determinants are and how evidence-based practices (EBPs) are sustained in tertiary settings (i.e., acute care hospitals). Current literature reveals several frameworks designed for implementation of EBPs (0–2 years), yet fewer exist for the sustainment of EBPs (>2 years) in clinical practice. Frameworks containing both phases generally list few determinants for the sustained use phase, but rather state ongoing monitoring or evaluation is necessary. Notably, a recent review identified six constructs and related strategies that facilitate sustainment, however, the pairing of determinants and how best to sustain EBPs in tertiary settings over time remains unclear. The aim of this paper is to present an evidence-informed framework, which incorporates constructs, determinants, and knowledge translation interventions (KTIs) to guide implementation practitioners and researchers in the ongoing use of EBPs over time.

**Methods:**

We combined the results of a systematic review and theory analysis of known sustainability frameworks/models/theories (F/M/Ts) with those from a case study using mixed methods that examined the ongoing use of an organization-wide pain EBP in a tertiary care center (hospital) in Canada. Data sources included peer-reviewed sustainability frameworks (*n* = 8) related to acute care, semi-structured interviews with nurses at the department (*n* = 3) and unit (*n* = 16) level, chart audits (*n* = 200), and document review (*n* = 29). We then compared unique framework components to the evolving literature and present main observations.

**Results:**

We present the Sustaining Innovations in Tertiary Settings (SITS) framework which consists of 7 unique constructs, 49 determinants, and 29 related KTIs that influence the sustainability of EBPs in tertiary settings. Three determinants and 8 KTIs had a continuous influence during implementation and sustained use phases. Attention to the level of application and changing conditions over time affecting determinants is required for sustainment. Use of a participatory approach to engage users in designing remedial plans and linking KTIs to target behaviors that incrementally address low adherence rates promotes sustainability.

**Conclusions:**

The SITS framework provides a novel resource to support future practice and research aimed at sustaining EBPs in tertiary settings and improving patient outcomes. Findings confirm the concept of sustainability is a “dynamic ongoing phase”.

## Introduction

Despite efforts among implementation practitioners and researchers a gap remains between efforts to embed evidence-based practices (EBPs), such as best practice guidelines (BPGs), in clinical practice and sustaining them over time beyond the initial implementation period (1). Ongoing discourse indicates conceptual frameworks are the best way to guide research and the implementation and sustainability of EBPs in clinical practice (2–6). To accomplish this, there are several published frameworks to choose from (4, 5). Specifically, many frameworks are designed for the implementation use phase of healthcare innovations (0–2 years) in clinical practice. However, few exist for the sustained use phase (7), especially for use within acute healthcare organizations, such as hospitals; hereafter referenced as tertiary settings. In this research, the sustained use of the evidence-based practice (EBP) change by users refers to maintaining ongoing EBP use, post an implementation period of greater than two years (i.e., >2 years) (8, 9). Distinctly, frameworks with combined implementation and sustainability constructs generally list fewer determinants for sustainability, or instead simply suggest ongoing monitoring or evaluation are necessary. As a result, practitioners and researchers alike must separately search the literature to identify sustainability determinants and related knowledge translation interventions (KTIs), (also referred to as strategies or approaches), known to influence use. Findings may or may not relate to the context of interest and often do not take into consideration the level of application (organizational verses unit level), nor the changing contextual influences over time. Measurably, this process is time consuming. This is particularly challenging to do in complex ever-changing contexts, such as in tertiary settings. There is a need for more comprehensive frameworks that combine both determinants and KTIs known to effectively facilitate the sustained use of EBPs to fill this gap in the literature and support practitioners and researchers working in clinical practice.

To date, evidence reveals the sustained use of an EBPs remains a persistent challenge in several settings (1, 10–13), and especially in tertiary settings (1, 14). In a recent empirical study that examined the determinants influencing ongoing use of EBPs in a multi-site hospital context over time, the impact of the changing underlying conditions on the determinants was revealed (15). The same study also presented insights related to the KTIs used to facilitate the sustained use of the EBP in clinical practice over time. These findings further articulated known strategies or approaches previously identified in a review by Lennox et al. (16) that included only 2 studies (out of 62) conducted in tertiary settings. These recent findings demonstrate that to promote healthcare innovation sustainability determinant identification is only part of the equation. Tailoring or linking KTIs to promote and “address specific determinants is the other critical step in the knowledge-to-action process” (2) to improve practice and related patient outcomes. This finding is not only relevant during the implementation phase but is an important component to consider during the sustained use phase for sustainability of EPBs in all contexts (17), including tertiary care settings. Currently, there are no frameworks which are explicit about the determinants and how related KTIs can be used to sustain EBPs in clinical practice during implementation (0–2 years) and sustained use phases (>2–10 years) (18) for clinical practice within tertiary settings.

The aim of this manuscript is to present a framework, which incorporates constructs, determinants, and related KTIs to guide implementation practitioners and researchers with the sustainability of EBPs, such as BPGs, in tertiary settings, namely acute care hospitals, to improve patient outcomes.

## Methods

### Design

To establish a framework to guide the sustainability of improved practice changes within tertiary settings, we focused our efforts on identifying relevant constructs, determinants, and related KTIs. Specifically, we combined the results of a case study using mixed methods that examined the ongoing use of an organization-wide Pain Best Practice Guideline (Pain BPG) in a hospital in Canada (15) with those from a recent systematic review and theory analysis of known sustainability frameworks/models/theories (F/M/Ts) relevant to acute care contexts (7). We compared the integrated findings with the evolving literature to confirm their inclusion in a comprehensive meta-synthesis of constructs, determinants, and related KTIs influencing sustainability for tertiary settings. The resultant ‘*Sustaining Innovations in Tertiary Settings (SITS) framework*’ is presented herein for ease of use by practitioners and researchers alike. We present main observations related to the *SITS framework* constructs, determinants and KTIs; discuss practice implications; outline strengths and limitations; and propose future directions. In conclusion, we highlight how the *SITS framework* contributes to the current knowledge base.

#### Inclusion criteria

In the systematic review and theory analysis (7), and the case study (15) only concepts or constructs, determinants and KTIs from known sustainability F/M/Ts and existing peer reviewed citations related to sustainability were included. Specifically, F/M/Ts needed to address the process of sustaining healthcare innovations, such as EBPs, in an acute clinical practice setting or an unspecified healthcare organization/setting. To be eligible, citations needed to be published in English; recommended for healthcare; and in a peer-reviewed journal. A citation was excluded if the F/M/T contained an implementation and sustainability F/M/T without an explicit breakdown of related sustainability determinants. Of note, this research was not designed to examine the influence of implementation on sustainability.

#### Sustainability definition

We used Moore et al.'s (3) definition of sustainability which states it “is a district concept that (1) occurs after a period of time; (2) the innovation or EBPs continues to be delivered; (3) and or individual behavior change (i.e., clinician, patient) is maintained; (4) the EBP and individual behavior change may evolve or adapt while; (5) continuing to produce benefits for individuals/systems” (3). The time period used to define the sustained use phase in this research is two years and beyond (>2 years.) which is congruent with current reviews (7–9, 14).

### Data sources

We first outline constructs, determinants, and related KTIs results from two key data sources: (i) systematic review and theory analysis results derived from known sustainability frameworks for acute care contexts (7), and (ii) synthesized case study findings for three timeframes: the implementation use phase (0–2 years), the sustained use phase (>2–10 years), and at the ten-year timeframe (15).

#### Systematic review and theory analysis

Eight sustainability F/M/Ts for acute care contexts included in the review (7) initially generated 152 sustainability determinants. Qualitative analysis revealed 37 core determinants, which are grouped into the following seven constructs: (1) *innovation*; (2) *adopter/user*; (3) *leadership and management*; (4) *inner context* (i.e., practice setting/organization); (5) *inner processes* (i.e., infrastructure processes, methods, systems, structures or strategies); (6) *outer context* or broader system determinants; and (7) *outcomes* consisting of descriptions without defined determinants, only definitions. Sixteen out of the 37 core determinants are identified as common, occurring in four or more F/M/Ts which are highlighted by single asterix (see Table 1).

**Table 1:** Synthesis of themes and determinants in known sustainability F/M/Ts for acute care (*N* = 8).

Theme /Concept	37 Core Factors	Unspecified setting Fwks					Acute care Fwks		
		1	2	3	5	6	4	7	8
**Innovation (** *Defined as: new process/change/ product/practice or program, innovation, intervention)*	**Relevance/consistent with competitive strategy (need)** *	✓	✓			✓		✓	
	**Characteristics (scale, shape & form, age, nature, type, integrity)** *	✓	✓		✓			✓	
	**Perceived centrality to organizational performance /platform /services** *	✓	✓		✓			✓	
	Fit with org's vision/mission, procedures/ strategies	✓		✓				✓	
	Adaptability of innovation			✓		✓		✓	
	**Benefits to patient, staff, organization (cost effective, efficiency & quality of care)** *		✓	✓	✓	✓		✓	
	Barrier Identification					✓			
**Adopters** *(Defined as: staff, stakeholder, user, adopter, actor, and or individual)*	Human resources—recruitment, processes, succession and leave planning (staffing)				✓	✓			
	**Individual commitment to innovation** *	✓	✓			✓		✓	
	**Individual competency (skill knowledge, absorptive capacity) to perform innovation** *	✓	✓		✓			✓	✓
	Internal cohesion between individual & commitment within the organization /stakeholder engagement leads to increased performance		✓					✓	✓
	Stakeholder Commitment to innovation			✓			✓		✓
	Stakeholder beliefs, attitude, perceptions, emotions, expectations towards innovation	✓		✓		✓			
	Champion presence & involvement					✓		✓	
**Leadership & Management** *(Defined as: style, approach, behaviors, engagement support, or feedback)*	**Management approach & engagement** *	✓	✓	✓	✓			✓	✓
	**Senior Leadership involvement & actions** *	✓	✓	✓				✓	
**Inner Context** *(Defined as: context, practice setting or organization)*	**Infrastructure support- Policies & Procedures based on Innovation** *	✓		✓				✓	✓
	Infrastructure support for innovation in job description with mechanism for recognizing achievement	✓		✓			✓		
	**Infrastructure support-equipment & supplies for innovation** *			✓			✓	✓	✓
	Organization—Absorptive capacity for innovation							✓	✓
	Cultural—Beliefs, values & perceptions to innovation	✓						✓	
	**Cultural—Climate** *	✓	✓		✓			✓	
	Cultural—innovation integrated into Norms (documents, protocols, manuals)	✓					✓		
	Political internal stakeholder coalition, power, influence	✓				✓		✓	
	Financial performance budgeting & measurement	✓				✓			
	Financial-internal funds & other non-financial resources of innovation					✓		✓	
**Processes** *(Defined as: processes, methods, systems, structures, or strategies)*	**Education & training processes** *			✓	✓	✓	✓	✓	
	Processual—Planning, method, & timing of embedding innovation	✓					✓	✓	
	**Processual- project structure & system to monitor/manage innovation** *	✓		✓	✓		✓	✓	
	**Organization—communication capacity for monitoring (reporting & feedback)** *	✓	✓	✓	✓	✓	✓	✓	
	Behavioural change strategies								✓
**Outer Context** *(Defined as: external condition, context, system, or environment)*	Soci-economic political threats, stability	✓			✓			✓	
	**External conditions, compatibility for innovation** *	✓	✓		✓			✓	
	Connection to broader external context		✓			✓		✓	
	External Support for innovation from Stakeholders	✓	✓					✓	
	**Political-Policy, legislation & Interests** *		✓		✓	✓		✓	
	Financial-external funds & other non-financial resources of innovation							✓	
**Outcomes** (*Defined as: outcomes, teamwork behaviors, consequences, or continuation of benefits)*	No factors explicitly defined in frameworks for this concept	✓				✓		✓	✓

1 = Buchanan SOCF, 2 = Racine MSI, 3 = Maher NHS SM, 4 = Slaghuis FMIS WP, 5 = Chambers DSF, 6 = Fox SITF, 7 = Fleiszer SIHF, 8 = Frykmann DCOMF.

* **Common Factors—**occurs in 4 or more F/M/Ts (7).

#### Case study

The case study (15) used an explanatory mixed method design to identify the 32 unique sustainability determinants and 29 related KTIs that influenced nurses ongoing use of an EBP; namely a Pain BPG, at the nursing department (an organizational perspective) and unit level (a point of care perspective) over three timeframes: (i) the implementation use phase (0–2 years), (ii) the sustained use phase over time (>2–10 years), and (iii) at the ten-year timeframe (see Table 2). Internal biannual audits revealed inpatient units demonstrated high to moderate adherence rates to several Pain BPG recommendations except those within the Medicine Care Department, necessitating further examination (15). Data sources included documents (*n* = 29), semi-structured interviews (*n* = 19), and inpatient chart audits (*n* = 200). Internal and external documents spanned the ten years (2007–2017). Responses from the three semi-structured department level interviews, were derived from nurses who worked across all 60 units over time. Documents and departmental findings were triangulated with unit level (subcases) quantitative results (e.g., audits) and qualitative findings (e.g., responses) derived from sixteen semi-structured unit nurse interviews.

**Table 2:** Case study findings for sustainability of EBPs in a tertiary setting.

DSF Themes/Constructs (19)	Integrated Determinants *N* = 32	*N* = 32 Unique Determinants			*N* = 29 Unique Knowledge Translation Interventions (KTIs0		
		Department RNs Implementation Determinants (0–2 years.) *n* = 3	Department RNs Sustainability Determinants (>2–10 years) *n* = 12	Unit RNs Sustainability Determinants (at 10 years) *n* = 31	Department RNs Implementation Phase (0–2 years.) KTIs (*n* = 12)	Department RNs Sustained Phase (>2–10 years.) KTIs (*n* = 22)	Unit RNs Sustained Phase (at 10 years.) KTIs (*n* = 11)
		3 ongoing Determinants			8 ongoing KTIs		
			2 + 8 Unique Determinants	2 + 19 Unique Determinants	+ 4 unique Imp KTIs	+ 14 unique Sust KTIs	+ 3 unique Sust KTIs
**Innovation** *(Defined as: new process/change/ product/practice or program, innovation, intervention)*	Relevance/consistent with competitive strategy (to addresses need/problem0✸	✓✸		✓✸			
	Adaptability of innovation	** **	** **	** **	**Embedding of Pain P/P** into existing unit processesù	**Embed ongoing refinements** into existing routine practices/processes &Pain P/P**ù**	**Routinize recommendations into nursing forms and practices/processes: e**mbed prompts**ù**
						** **	**Digitalized Pain P/P and forms** into new eHealth record
		** **	** **	** **	**Pain P/P established Interdisciplinary** for all disciplines		
	Benefits to patient, staff, organization (cost effective, efficiency & quality of care)			✓			
	Barrier Identification			** **	**Use frameworks** to guide implementation and identify barriers		
**Practice Setting** *(Defined as inner context)*	Human resources—recruitment, processes, succession and leave planning (staffing/compliment)	** **	✓	** **	**Secure internal financial** commitment—time and Human resources to participate on cttees & to implement KTIs		
	Student turnover (medical)	** **	✓	** **	** **		
	Individual commitment to innovation			✓			
	Individual competency (skill knowledge, absorptive capacity) to perform innovation and time management to use innovation			✓			
	expert consultants /resources			✓			
	Internal cohesion btwn individual & commitment within the organization /stakeholder engagement leads to increased performance (senior nurse mentors /influencers verses Clinical Care Leaders))			✓			**Mentorship used by senior nurses t**o support Pain P/P use:
	Stakeholder Commitment to innovation (IP—interprofessional)	** **	** **	✓	**Joint collaboration** of human resources **from all levels of nursing** plus other disciplines to develop departmental implementation plan**ù**		**Engages IP stakeholder involvement:** all professions to follow policy participate on cttees**ù**
	Stakeholder beliefs, attitude, perceptions, emotions, expectations towards innovation and user motivation/resistance		✓	✓			
	Population characteristic/needs/acuity level			✓			
	Users awareness / familiarity with innovation			✓			
**Practice Setting** *(Defined as inner context)*	**Leadership commitment (dept level)** ✸	✓✸	✓✸	✓✸	**Formalize BPG Coordinator role** **ù**	**Comparing survey results among units created a sense of competition** among leaders and users to improve**ù**	**Leadership strategiesù** - Clinical Coordinator—dept level: (support for big issues during shifts) - Clinical Care Leaders—unit level (get involved in unit level issues to support ongoing improvements) - Unit Managers—unit level (get involved in unit wide issues, help with remedial action plans to reinforce target behaviors, review incidents, encourages education training)
	Management approach & engagement (commitment unit level)		✓	✓			
	Senior Leadership involvement & actions		✓				
**Practice Setting** *(Defined as inner context)*	Infrastructure support- Policies & Procedures based on Innovation (i.e., cttees, key people in nursing dept– i.e. educators, champions, NPP reps)			✓			
	Infrastructure support for innovation in job description with mechanism for recognizing achievement					**Performance Evaluation indicators** for monitoring rt innovation = leaders, managers, and staff	
	Infrastructure support-equipment & supplies for innovation (and resources = pamphlets)			✓			
	Physical layout/structure of wards			✓			
	Competing corporate priorities		✓				
	Cultural—Beliefs, values & perceptions to innovation			✓			
	Cultural—Climate (doing research)			✓			
	Cultural—innovation integrated into Norms (documents, protocols, manuals)			✓		**Unit leaders lead dept and unit level patient centered initiatives for pain care based on unit routine practices** -with adoption of EBP care	
	Team culture embraces innovation		** **	✓	**Obtaining buy-in and Formalize nurse leaders’ involvement** on Steering Cttee**ù**	**Corporate level Internal cttees’ support ongoing review of clinical tactics** support sustained use ie Patient Experience Steering cttee and Accreditation workgroup**ù**	**Fostering an IP and EBP culture** among IP team to support Pain P/P use:**ù**
	Political internal stakeholder coalition, power, influence					**Dept determine EBP priorities**	
	Financial performance budgeting & measurement			** **	** Secure external fundsù** a) RNAO PBSO—secure operating funds for initial training and resource s to build capacity b) secure capital external financial support—for point of care surveying system	**Development of an electronic monitoring system** to measure nursing sensitive indicators provide monitoring of BPG adherence**ù**	
**Practice Setting** *(Defined as inner context)*	Workload /staffing patterns			✓			
	Education & training processes	** **	** **	** **	**Pain Council established—Interdisciplinary taskforce** leads initial policy development, education strategies and future policy revision**ù**	**NPP reps develop formal and informal education** initiatives at dept and unit level in 2014 initially performed by the Pain Council.**ù**	**Ongoing Education** to support Pain P/P use by NPP and Educators: - education days,: - mandatory online modules: - updates, refreshers, seminars**ù**
		** **	** **	** **	**Educating Champions** –to be clinical experts on units, with APNs:**ù**	**Trains 170 Unit level expertise** to support use of Pain P/P s = Champions, educators, APNs, work across units as clinical resource**ù**	**Ongoing Training** to support Pain P/P use by NPP and Educators: - general hospital orientation,: - −1 on 1 training, in-services, solve recurrent problems**ù**
						**Ongoing pain care education** support at dept and unit levels becomes tailored over time ie 1 on 1, case studies	
		** **	** **	** **	** **	Mandatory **eLearn training** system	
		** **	** **	** **	** **	**Unit specific training** of staff provided **based on audit remedial action plans** to improve on related BPG survey indicators	
		** **	** **	** **	** **	Develop unit specific **additional resources/tools** over time	
**Practice Setting** *(Defined as inner context)*	Processual—Planning, method, & timing of embedding innovation			✓	Use **multi-modal approach to disseminate**		
	Processual- project structure & system to monitor/manage innovation	** **	** **	** **	** **	**Spread EBP** to additional areas	
		** **	** **	** **	**Established Pain BPG taskforce/workgroup** in NPP dept—enduring central reporting and monitoring structure for ongoing implementation and evaluation**ù**	**NPP and Unit Leaders facilitate/lead remedial action plan for under performing units** **ù**	**Monitoring and evaluation:** Dept level—ongoing training to do survey Unit level—audit and feedback provided (timely sharing of audit data, focuses biannual audit questions on target behaviors) Unit level—Patient satisfaction survey results shared reviews incidents and develop strategies to prevent them in staff mtgs**ù**
	Organization—communication capacity for monitoring (reporting & feedback)	** **	** **	✓	** **	Ongoing biannual **training** of staff **to conduct prevalence survey**	
		** **	** **	** **	** **	**NPP Establishes regular performance monitoring:** includes results from biannual prevalence audit and internal incident reporting	
		** **	** **	** **	** **	Timely reporting of prevalence survey results led to **course correcting changes**	
	Formal communicating/reporting systems for client info between practitioners (documented)			✓			Establishing effective communications between providers, reporting practices—bedside exchange, whiteboards, clipboards
**Broader system** (*Defined as: external condition, context, system, or environment*)	External conditions, compatibility for innovation (consumer demand)		✓				
	**External pressure/demand (e.g., professional/regulatory bodies, Ministry, funding bodies)** ✸	✓✸	✓✸	✓✸		**New evidence released—**Integrating into BPG and ongoing education	
**Broader system** (*Defined as: external condition, context, system, or environment*)	Connection to broader external context (regional, national, international links)		✓			**## Staff participation on a regional network—**provide access to new research and related outcomes for pain management	
	External Support for innovation from Stakeholders (recognition)		✓			**Benchmarking** to external sources best practices	
	Goal Alignment with external agencies (e.g., Education institutes)		✓				

ï Determinants common over three timeframes—Implementation phases (0–2 years), Sustained use phases (2–10 years, and at 10 years).

ù KTIs common over three timeframes– Implementation phases (0–2 years), Sustained use phases (2–10 years, and at 10 years).

All sustainability determinants (*N* = 32) and related KTIs (*N* = 29) influencing Pain BPG use over time were grouped into 3 constructs guided by the Dynamic Sustainability Framework (DSF) (19): the ‘Innovation’, ‘Practice Setting’, and ‘Broader System’ constructs. Together, department and unit level nurses identified 3 out of the 32 determinants (i.e., perceived *need*, *leadership commitment*, *external demand*) that continuously influenced sustained use over all three time periods. Notably, these three determinants were identified in different constructs: perceived *need* within the ‘Innovation’ construct, *leadership commitment* within the ‘Practice Setting’ construct, and *external demand* within the ‘Broader System’ construct. Department and unit nurses further identified two determinants (e.g., *stakeholder engagement*, unit level *management commitment*) that influenced ongoing use for both sustained use phase timeframes (e.g., >2–10 years, at 10 years.). Department level nurses uniquely identified eight more determinants for the sustained use phase (>2–10 years), and unit nurses uniquely identified an additional 19 determinants for the ten-year period detailed on Table 2.

Among the 29 KTIs identified within the case study, department and unit nurses described 8 KTIs that continuously promoted sustained Pain BPG use over all three time periods. These eight KTIs are within the DSF ‘Innovation’ and ‘Practice Setting’ constructs (19). Specifically, the first KTI: e*mbedding of recommendations* and *ongoing refinements* into existing forms and processes (i.e., integrating *prompts* into formal documentation processes and routine practices) facilitated high adherence rates. Second KTI: engaging *stakeholder joint collaboration* from the start, on all levels [e.g., consulting with interprofessional (IP) team members on the BPG] promoted use of EBPs among all disciplines. Third KTI: *formalizing the supervision of BPGs* within the Nursing Professional Practice (*N*PP) center and in related job descriptions for NPP leaders (e.g., BPG Coordinator and NPP department level representatives) provided an enduring centralized infrastructure to support ongoing BPG implementation, monitoring and reporting efforts over time. Fourth KTI: *obtaining buy-in* and *formalizing nursing leaders' involvement on committees* to support clinical tactics to sustain use of the innovation fostered leadership's commitment to evidence-based practice and culture among team members. Fifth KTI: *securing financial funds* externally and internally to develop a software system to monitor BPG nursing sensitive indicators at point of care facilitated BPG use beyond implementation. Sixth KTI: providing *ongoing education and training* support through formal and informal initiatives, on all levels, promoted evidence-based practice among new recruits and senior staff nurses. Seventh KTI: *educating and training champions* over time ensured access to unit level BPG expertise promoting sustained use of BPG recommendations. Eight KTI: *establishing a central reporting and monitoring structure* within the NPP department facilitated timely feedback of ongoing prevalence audit results to units and reporting of remedial action plans designed to address low adherence rates.

Additionally, department level nurses uniquely identified four KTIs for the implementation use phase (0–2 years), and fourteen KTIs for the sustained use phase (>2–10 years) (see Table 2). Unique implementation use phase (0–2 years.) KTIs used included: (i) establishing an interdisciplinary Pain policy/protocol; (ii) using a framework to guide implementation and to identify barriers; (iii) securing internal financial commitment; and (iv) using a multi-modal approach to disseminate the Pain BPG across all units. During the sustain use phase (>2–10 years.) department nurses identified the following 14 unique KTIs that promoted Pain BPG use over time: (i) establishing performance evaluation indicators related to the Pain BPG for unit leaders; (ii) having unit leaders lead department and unit level pain care initiatives; (iii) encouraging unit leaders to determine EBP priorities; (iv) having unit leaders facilitate ongoing related education tailored to units; (v) implementing mandatory elearn training related to BPGs; (vi) providing unit specific training of staff based on audit remedial action plans to improve BPG survey indicators; (vii) developing additional unit specific BPG resources/tools: (viii) spreading the Pain BPG to outpatient units; (ix) offering ongoing biannual training of staff to conduct prevalence surveys; (x) requiring leaders to formally report unit performance monitoring related to BPGs; (xi) developing remedial action plans in response to timely prevalence reports; (xii) integrating new evidence into BPG and ongoing education initiatives; (xiii) encouraging staff participation on regional networks; and (xiv) benchmarking performance to external sources and best practices.

Unit level nurses further identified three KTIs unique to the ten-year timeframe (see Table 2). Specifically, unit nurses indicated (i) digitalizing or embedding recommendations from the Pain Policy/protocol into the eHealth record; (ii) mentorship by senior nurses; and (iii) effective communication and reporting practices between providers influenced their sustained use of the Pain BPG. Notably, unit level audit findings reportedly demonstrated ‘Innovation’ and ‘Practice Setting’ KTIs designed to standardize and monitor nursing documentation practices over time effectively promoted ongoing EBP use over time (15).

### Analysis

Qualitative content analysis (20) was conducted to identify the total number of unique constructs, determinants and KTIs among the key data sources. Initially, we deductively mapped the three constructs, determinants and related KTIs identified in the empirical case study (15) to the seven constructs synthesized from theoretical conceptualizations of the eight sustainability frameworks included in the systematic review (7). We then inductively triangulated the determinants and related KTIs from the case study with the determinants identified within the systematic review, removing duplicates, and maintaining alignment or grouping within the seven constructs. Determinants identified in the case study, not previously identified within the synthesis of the eight F/M/Ts, were then examined by comparing them with those identified in two recent reviews related to sustainability (1, 21). Finally, all 29 KTIs derived from the case study (15) were compared with the current literature (16) to examine similarities and differences. Lastly, we present main observations related to the resultant synthesis of constructs, determinants and KTIs, which formed the ‘*Sustaining Innovations in Tertiary Settings*’ (SITS) framework.

## Results

### Combined results for tertiary settings

**Figure 1:**
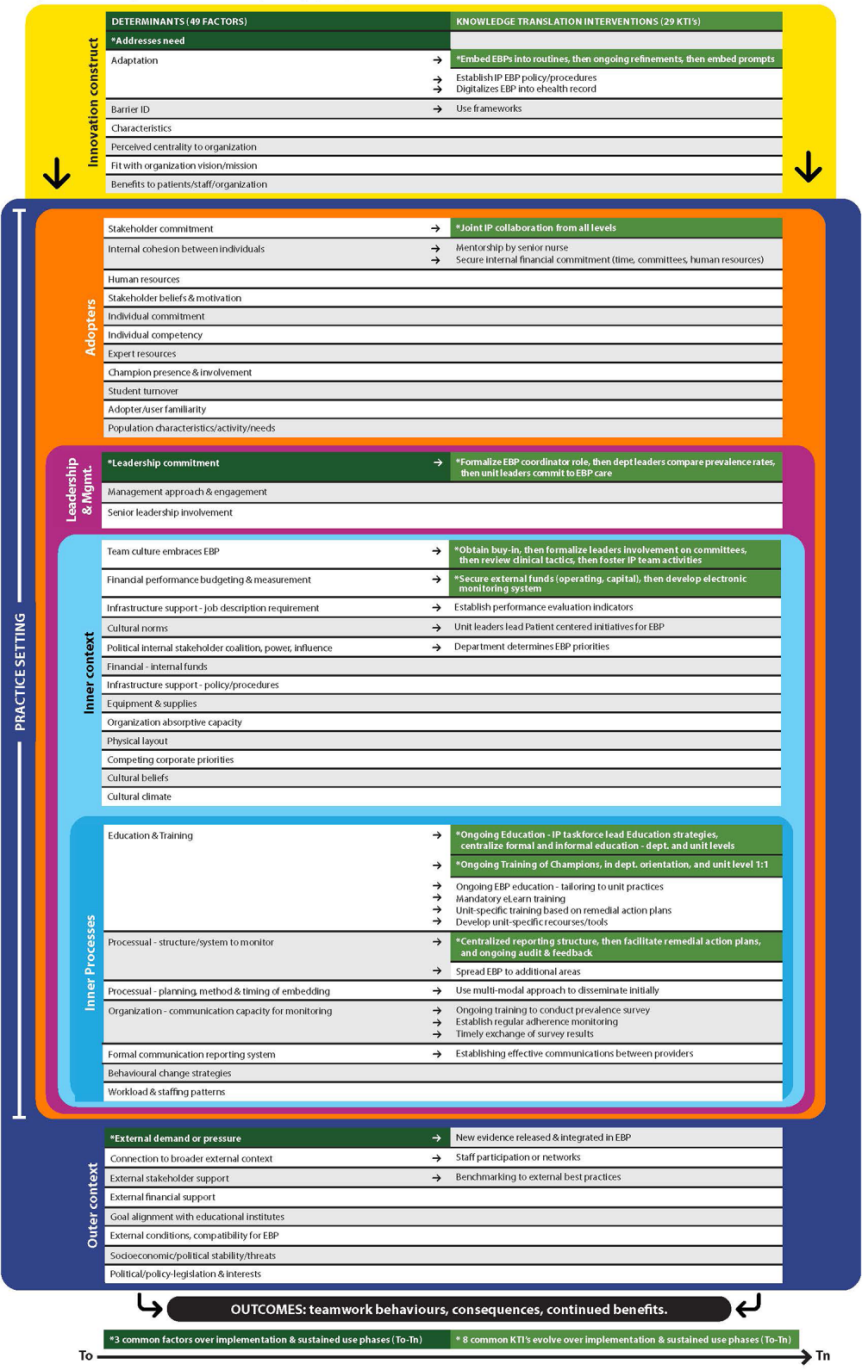
Sustaining innovations in tertiary settings (SITS) framework.

Qualitative content analysis and triangulation of the constructs or concepts, determinants and related KTIs from the case study (15) and the systematic review (7) revealed a comprehensive meta-synthesis of 7 unique constructs, 49 unique sustainability determinants, and 29 related KTIs (see Table 3). We present our comparison of these integrated findings to the evolving literature to confirm inclusion within the new framework, entitled ‘*Sustaining Innovations in Tertiary Settings*’ (SITS) (see Figure 1, and Table 4 for details).

**Table 3:** Combined findings for sustaining innovations in tertiary settings.

Dynamic Sustainability Framework (DSF) Constructs (19)	7 Themes/Constructs (**7**)	Determinants (*N* = 49)	Unspecified setting Fwks					Acute care Fwks			Imp Factors (0–2 years.) *n* = 3	Sust Factors (>2–10 years) *n* = 12	Sust Factors (at 10 years) *n* = 31	Department RNs Implementation (0–2 years.) 8 + 4 KTIs (*n* = 12)	Department RNs Sustainability (>2–10 years.) 8 + 14 KTIs (*n* = 22)	Unit RNs Sustainability (at 10 years.) 8 + 3 KTIs (*n* = 11)
			1	2	3	5	6	4	7	8						
**Innovation** *(Defined as: new process/change/ product/practice or program, innovation, intervention*	**Innovation** *(defined as: new process/change/ product/practice or program, innovation, intervention)*	Relevance/consistent with competitive strategy (to addresses need/problem*	✓	✓			✓		✓		✓**✸**	✓**✸**				
		Characteristics (scale, shape & form, age, nature, type, integrity)*	✓	✓		✓			✓							
		Perceived centrality to organizational performance /platform /services*	✓	✓		✓			✓							
		Fit with org's vision/mission, procedures/ strategies	✓		✓				✓							
		Adaptability of innovation			✓		✓		✓		** **	** **	** **	**Embedding of Pain P/P** into existing unit processes**ù**	**Embed ongoing refinements** into existing routine practices/processes & Pain P/P**ù**	**Routinize recommendations into nursing forms and practices/processes: e**mbed prompts**ù**
															** **	**Digitalized Pain P/P and forms** into new eHealth record
											** **	** **	** **	**Pain P/P established Interdisciplinary** for all disciplines		
		Benefits to patient, staff, organization (cost effective, efficiency & quality of care)*		✓	✓	✓	✓		✓				✓			
		Barrier Identification					✓						** **	**Use frameworks** to guide implementation and Id barriers		
**Practice Setting** *(Defined as inner context)*	**Adopters** *(defined as: staff, stakeholder, user, adopter, actor, and or individual)*	Human resources—recruitment, processes, succession and leave planning (staffing/compliment)				✓	✓				** **	✓	** **	**Secure internal financial** commitment—time and Human resources to participate on cttees and to implement KTIs		
		**Student turnover (medical)** **									** **	✓	** **	** **		
		Individual commitment to innovation*	✓	✓			✓		✓				✓			
		Individual competency (skill knowledge, absorptive capacity) to perform innovation and time management to use innovation*	✓	✓		✓			✓	✓			✓✓			
		**expert consultants /resources** **											✓			
		Internal cohesion btwn individual & commitment within the organization /stakeholder engagement leads to increased performance (senior nurse mentors /influencers)		✓					✓	✓			✓			**Mentorship used by senior nurses t**o support Pain P/P use:
		Stakeholder Commitment to innovation			✓			✓		✓	** **	** **	✓✓✓✓	**Joint collaboration** of human resources **from all levels of nursing** plus other disciplines to develop departmental implementation plan**ù**		**Engages IP stakeholder involvement:** all professions to follow policy participate on cttees**ù**
		Stakeholder beliefs, attitude, perceptions, emotions, expectations towards innovation and user motivation/resistance	✓		✓		✓					✓	✓✓			
		**Population characteristic/needs/acuity level** **											✓			
		**Users awareness / familiarity with innovation** **											✓			
		Champion presence & involvement					✓		✓							
**Practice Setting** *(Defined as inner context)*	**Leadership & Management** *(defined as: style, approach, behaviors, engagement support, or feedback)*	**leadership commitment (dept level)** **									✓**✸**	✓**✸**	** **	**Formalize BPG Coordinator role** **ù**	**Dept Leaders Comparing survey results** among units created a sense of competition among leaders and users to improve**ù**	**Leadership strategies****ù** - Clinical Coordinator- dept level: (support for big issues during shifts) - Clinical Care Leaders—unit level (get involved in unit level issues to support ongoing improvements) - Unit Managers—unit level (get involved in unit wide issues, help with remedial action plans to reinforce target behaviors, review incidents, encourages education training)
		Management approach & engagement (commitment unit level)*	✓	✓	✓	✓			✓	✓		✓**✸**	✓✓✓**✸**			
		Senior Leadership involvement & actions*	✓	✓	✓				✓			✓				
	**Inner Context** *(defined as: context, practice setting or organization)*	Infrastructure support- Policies & Procedures based on Innovation*	✓		✓				✓	✓			✓			
		Infrastructure support for innovation in job description with mechanism for recognizing achievement	✓		✓			✓							**Performance Evaluation indicators** for monitoring rt innovation = leaders, managers, and staff	
		Infrastructure support-equipment & supplies for innovation (and resources = pamphlets)*			✓			✓	✓	✓			✓			
		Organization—Absorptive capacity for innovation							✓	✓		** **				
		**Physical layout/structure of wards** **											✓			
		**Competing corporate priorities** **										✓				
		Cultural—Beliefs, values & perceptions to innov	✓						✓				✓			
		Cultural—Climate*	✓	✓		✓			✓				✓			
		Cultural—innovation integrated into Norms (documents, protocols, manuals)	✓					✓					✓		**Unit leaders lead dept and unit level patient centered initiatives for pain care based on unit routine practices** -with adoption of EBP care	
		**Team culture embraces innovation** **										** **	✓	**Obtaining buy-in and Formalize nurse leaders’ involvement** on Steering Cttee**ù**	**Corporate level Internal cttees’ support ongoing review of clinical tactics** support sustained use ie Patient Experience Steering cttee and Accreditation workgroup**ù**	**Fostering an IP and EBP culture** among IP team to support Pain P/P use:**ù**
		Political internal stakeholder coalition, power, influence	✓				✓		✓						**Dept determine EBP priorities**	
		Financial performance budgeting & measurement	✓				✓						** **	** Secure external funds****ù** a) RNAO PBSO—secure operating funds for initial training and resource s to build capacity b) secure capital external financial support—for point of care surveying system	**Development of an electronic monitoring system** to measure nursing sensitive indicators provide monitoring of BPG adherence**ù**	
		Financial-internal funds & other non-financial resources of innovation					✓		✓							
**Practice Setting** *(Defined as inner context)*	**Inner Processes** *(defined as processes, methods, systems, in the inner environment)*	**workload /staffing patterns** **											✓			
		Education & training processes*			✓	✓	✓	✓	✓		** **	** **	** **	**Pain Council established—Interdisciplinary taskforce** leads initial policy development, education strategies and future policy revision**ù**	**NPP reps develop formal and informal education** initiatives at dept and unit level in 2014 initially performed by the Pain Council.**ù**	**Ongoing Education** to support Pain P/P use by NPP and Educators:**ù** - education days, - mandatory online modules - updates, refreshers, seminars
											** **	** **	** **	**Training/Educating Champions** –to be clinical experts on units, with APNs	**Trains 170 Unit level expertise** to support use of Pain P/P s = Champions, educators, APNs, work across units as clinical resource	**Ongoing Training** to support Pain P/P use by NPP and Educators: - general hospital orientation, - 1 on 1 training, in-services, solve recurrent problems
															**Ongoing pain care education** support at dept and unit levels becomes tailored overtime ie 1 on 1, case studies	
											** **	** **	** **	** **	Mandatory **eLearn training** system	
											** **	** **	** **	** **	**Unit specific training** of staff provided **based on audit remedial action plans** to improve on related BPG survey indicators	
											** **	** **	** **	** **	Develop unit specific **additional resources/tools** overtime	
		Processual—Planning, method, & timing of embedding innovation	✓					✓	✓				✓	Use **multi-modal approach to disseminate**		
		Processual- project structure & system to monitor/manage innovation*	✓		✓	✓		✓	✓		** **	** **	** **	** **	**Spread EBP** to additional areas	
											** **	** **	** **	**Established Pain BPG taskforce/workgroup** in NPP dept—enduring central reporting and monitoring structure for ongoing implementation and evaluation**ù**	**NPP and Unit Leaders facilitate/lead remedial action plan for under performing units** **ù**	**Monitoring and evaluation:****ù** Dept level—ongoing training to do survey Unit level—audit and feedback provided (timely sharing of audit data, focuses biannual audit questions on target behaviors) Unit level—Patient satisfaction survey results shared reviews incidents and develop strategies to prevent them in staff mtgs
		Organization—communication capacity for monitoring (reporting & feedback)*	✓	✓	✓	✓	✓	✓	✓		** **	** **	✓	** **	Ongoing biannual **training** of staff **to conduct prevalence survey**	
											** **	** **	** **	** **	**NPP Establishes regular performance monitoring:** includes results from biannual prevalence audit and internal incident reporting	
											** **	** **	** **	** **	Timely reporting of prevalence survey results led to **course correcting changes**	
		**Formal communicating/reporting systems for client info btwn practitioners (documented)** **											✓✓			Establishing effective communications between providers, reporting practices—bedside exchange, whiteboards, clipboards
		Behavioural change strategies								✓						
**Broader system** (*Defined as: external condition*, *context, system, or environment)*	**Outer Context** (defined as: external condition, context, system, or environment)	Soci-economic political threats, stability	✓			✓			✓							
		External conditions, compatibility for innovation (consumer demand)*	✓	✓		✓			✓			✓				
		**External pressure/demand (e.g., professional/regulatory bodies, Ministry, funding bodies)** **									✓**✸**	✓**✸**	✓		**New evidence released—**Integrating into BPG and ongoing education	
		Connection to broader external context (regional, national, international links)		✓			✓		✓			✓			**## Staff participation on a regional network—**provide access to new research and related outcomes for pain management	
		External Support for innovation from Stakeholders (recognition)	✓	✓					✓			✓			**Benchmarking** to external sources best practices	
		**Goal Alignment with external agencies (e.g., Education institutes)** **										✓				
		Political-Policy, legislation & Interests*		✓		✓	✓		✓							
		Financial-**external** funds & other non-financial resources of innovation							✓							
**Outcomes** (*Defined as the continuation of intended benefits*)	**Outcomes** (defined as: outcomes, teamwork behaviors, consequences, or continuation of benefits)	No factors explicitly defined in frameworks for this concept	✓				✓		✓	✓						

1 = Buchanan SOCF, 2 = Racine MSI, 3 = Maher NHS-SM, 4 = Slaghuis FMIS-WP, 5 = Chambers DSF, 6 = Fox SITF, 7 = Fleiszer SIHF, 8 = Frykmann DCOMF.

* **Common Determinants—**occurs in 4 or more F/M/Ts from systematic review (7).

** **12 Sustainability determinants—**additions from case study (15).

ï **3 Common Determinants** over three timeframes—Implementation phases (0–2 years), Sustained use phases (2–10 years, and at 10 years).

ù **8 Common KTIs** over three timeframes—Implementation phases (0–2 years), Sustained use phases (2–10 years, and at 10 years).

**Table 4:** The sustaining innovations in tertiary settings (SITS) framework.

7 Constructs	49 Unique Sustainability Determinants	29 Unique Sustainability-orientated Knowledge Translation Interventions (KTIs)		
(*N* = 7)	(*N* = 49)	Department Level Implementation Phase KTIs (0–2 years.) (*N* = 8 + 4)	Department Level Sustainability Phase KTIs (>2–10 years.) (*N* = 8 + 14)	Unit Level Sustainability Phase KTIs (at 10 years.) (*N* = 8 + 3)
**Innovation** *(defined as: new process/change/ product/practice or program, innovation, intervention)*	Relevance/consistent with competitive strategy (addresses **NEED or** problem)✸			
	Adaptability of innovation	**ùEmbedding of EBP** into existing unit processes	**ùEmbed ongoing refinements** into existing routine practices/processes	**ùRoutinize recommendations into nursing forms and practices/processes: embed prompts**
			** **	**Digitalized EBP and forms** into new eHealth record
		**Established Interdisciplinary EBP** policy/procedure for all disciplines		
	Barrier Identification	**Use frameworks** to guide implementation and identify barriers		
	Characteristics (scale, shape & form, age, nature, type, integrity)			
	Perceived centrality to organizational performance /platform /services			
	Fit with org's vision/mission, procedures/ strategies			
	Benefits to patient, staff, organization (cost effective, efficiency & quality of care)			
**Adopters** *(defined as: staff, stakeholder, user, adopter, actor, and or individual)*	Stakeholder Commitment to innovation	**ùJoint collaboration** of human resources **from all levels of nursing** plus other disciplines to develop departmental implementation plan		**ùEngages IP stakeholder involvement:** all professions to follow policy participate on cttees
	Internal cohesion between individual & commitment within the organization /stakeholder engagement leads to increased performance (senior nurse mentors /influencers)	** **	** **	**Mentorship used by senior nurses t**o support EBP use:
	Human resources—recruitment, processes, succession and leave planning (staffing/compliment)	**Secure internal financial** commitment—time and Human resources to participate on cttees and to implement KTIs		
	Stakeholder beliefs, attitude, perceptions, emotions, expectations towards innovation and user motivation/resistance			
	Individual commitment to innovation			
	Individual competency (skill knowledge, absorptive capacity) to perform innovation and time management to use innovation			
	expert consultants /resources**			
	Champion presence & involvement			
	Student turnover (medical)**			
	Users awareness / familiarity with innovation**			
	Population characteristic/needs/acuity level**			
**Leadership & Management** *(defined as: style, approach, behaviors, engagement support, or feedback)*	**Leadership commitment (dept level)**✸,**	**ùFormalize EBP Coordinator role**	**ù Dept Leaders Comparing survey results** among units created a sense of competition among leaders and users to improve	**ùLeadership strategies** - Clinical Coordinator- dept level: (support for big issues during shifts) - Clinical Care Leaders—unit level (get involved in unit level issues to support ongoing improvements) - Unit Managers—unit level (get involved in unit wide issues, help with remedial action plans to reinforce target behaviors, review incidents, encourages education training)
	Management approach & engagement (commitment unit level)			
	Senior Leadership involvement & actions			
**Inner Context** *(defined as: context, practice setting or organization)*	Team culture embraces innovation**	**ùObtaining buy-in and Formalize nurse leaders’ involvement** on Steering Cttee	**ùCorporate level Internal cttees’ support ongoing review of clinical tactics** support sustained use ie Patient Experience Steering cttee and Accreditation workgroup	**ùFostering an IP and EBP culture** among IP team to support EBP use:
	Financial performance budgeting & measurement	**ù Secure external funds** a) RNAO PBSO—secure operating funds for initial training and resource s to build capacity b) secure capital external financial support—for point of care surveying system	**ùDevelopment of an electronic monitoring system** to measure nursing sensitive indicators provide monitoring of EBP adherence	
	Infrastructure support for innovation in job description with mechanism for recognizing achievement—requirement		**Performance Evaluation indicators** for monitoring rt innovation = leaders, managers, and staff	
	Cultural—innovation integrated into Norms (documents, protocols, manuals)		**Unit leaders lead dept and unit level patient centered initiatives for EBP based on unit routine practices**	
	Political internal stakeholder coalition, power, influence		**Depts determine EBP priorities**	** **
	Financial-internal funds & other non-financial resources of innovation			
	Infrastructure support- Policies & Procedures based on Innovation			
	Infrastructure support-equipment & supplies for innovation (and resources = pamphlets)			
	Organization—Absorptive capacity for innovation			
	physical layout/structure of wards**			
	competing corporate priorities**			
	Cultural—Beliefs, values & perceptions to innovation			
	Cultural—Climate			
**Inner Processes** *(defined as processes, methods, systems in the inner environment)*	Education & training processes	**ùPain Council established—Interdisciplinary taskforce** leads initial policy development, education strategies and future policy revision	**ùNPP reps develop formal and informal education** initiatives at dept and unit level in 2014 initially performed by the Pain Council.	**ùOngoing Education** to support EBP use by NPP and Educators: - education days, - mandatory online modules - updates, refreshers, seminars
		**ùTraining/Educating Champions** –to be clinical experts on units, with APNs	**ùTrains 170 Unit level expertise** to support use of EBP s = Champions, educators, APNs, work across units as clinical resource	**ùOngoing Training** to support EBP use by NPP and Educators: - general hospital orientation, - 1 on 1 training, in-services, solve recurrent problems
			**Ongoing EBP education** support at dept and unit levels becomes tailored overtime i.e., 1 on 1, case studies	
		** **	Mandatory **eLearn training** system	
		** **	**Unit specific training** of staff provided **based on audit remedial action plans** to improve on related EBP survey indicators	
		** **	Develop unit specific **additional resources/tools** overtime	
	Processual- project structure & system to monitor/manage innovation	**ùEstablished EBP taskforce/workgroup** in NPP dept—enduring central reporting and monitoring structure for ongoing implementation and evaluation	**ùNPP and Unit Leaders facilitate/lead remedial action plan for under performing units**	**ùMonitoring and evaluation:** Dept level—ongoing training to do survey Unit level—audit and feedback provided (timely sharing of audit data, focuses biannual audit questions on target behaviors) Unit level—Patient satisfaction survey results shared reviews incidents and develop strategies to prevent them in staff mtgs
		** **	**Spread EBP** to additional areas	
	Processual—Planning, method, & timing of embedding innovation	Use **multi-modal approach to disseminate**		
	Organization—communication capacity for monitoring (reporting & feedback)	** **	Ongoing biannual **training** of staff **to conduct prevalence survey**	
		** **	**NPP Establishes regular performance monitoring:** includes results from biannual prevalence audit and internal incident reporting	
		** **	Timely reporting of prevalence survey results led to **course correcting changes**	
	Formal communicating/reporting systems for client info between practitioners (documented)**			Establishing effective communications between providers, reporting practices—bedside exchange, whiteboards, clipboards
	Behavioural change strategies			
	workload /staffing patterns**			
**Outer Context** (defined as: external condition, context, system, or environment)	**External pressure/demand (e.g., professional/regulatory bodies, Ministry, funding bodies)**✸**		**New evidence released—**Integrating into EBP and ongoing education	
	Connection to broader external context (regional, national, international links)		**## Staff participation on a regional network—**provide access to new research and related outcomes for pain management	
	External Support for innovation from Stakeholders (recognition)		**Benchmarking** to external sources best practices	
	Financial-**external** funds & other non-financial resources of innovation			
	Goal Alignment with external agencies (e.g., Education institutes)**			
	External conditions, compatibility for innovation (consumer demand)			
	Soci-economic political threats, stability			
	Political-Policy, legislation & Interests			
**Outcomes** (defined as: outcomes, teamwork behaviors, consequences, or continuation of benefits)	No factors explicitly defined in frameworks for this concept			

**12 Sustainability Determinants- additions from the case study (15).

ï 3 Common Determinants over three timeframes—Implementation phase (0–2 years), Sustained use phase (2–10 years, and at 10 years).

ù 8 Common KTI over three timeframes—Implementation phase (0–2 years), Sustained use phase (2–10 years, and at 10 years).

### Determinants influencing sustainability in tertiary settings

**Figure 2:**
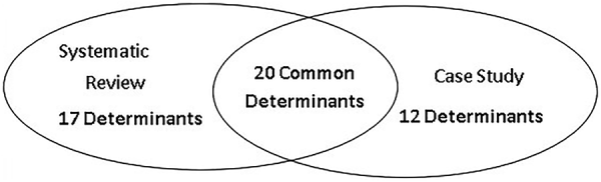
Diagram of the 49 unique sustainability determinants for tertiary settings.

Examination of the 49 determinants revealed 20 common sustainability determinants between the systematic review (7) and case study results (15), 17 determinants unique to the systematic review, and 12 determinants unique to the case study. All 49 sustainability determinants aligned with 6 (of the 7) constructs identified in the systematic review (7) (see Figure 2). Notably, no determinants were reported for the ‘Outcome’ construct in the case study (15). This is not unexpected given ‘Outcomes’ is not identified as a construct within the DSF (19), but instead defined as “the continuation of intended benefits” (19), a finding previously noted (7, 22).

The 17 sustainability determinants previously identified in the systematic review (7) did not align with those in the case study (15). This finding is not surprising, given the case study only used one of the frameworks; namely the DSF (19), included in the systematic review to guide data collection and analysis (15). As such, the DSF did provide the same comprehensive list of determinants provided in the results of the systematic review (7). Furthermore, our review of the case study data collection tools indicated no specific questions were used related to the 17 determinants. Thus, we cannot say with any definitiveness whether the 17 determinants were present (or not) in the case study (15). However, this does demonstrate not all determinants apply every time in all real-world settings.

The remaining 12 sustainability determinants, uniquely identified in the case study (15), lie within the five ‘context constructs’ identified in the systematic review (e.g., *Adopters, Leadership & Management, Inner Context, Inner Processes, Outer Context*) (7), and those previously reported in the evolving literature related to sustainability of EBPs in healthcare settings (1, 21). Specifically, the 12 determinants align with the ‘domains, attributes and related features of context’ influencing the use of EBPs in research and clinical practice identified in a recent review and concept analysis of context by Squires et al. (21) and the ‘emerging contextual influences’ impacting sustainability identified in another review by Shelton et al. (1).

#### Construct/theme similarities in the literature categorizing the twelve determinants

We present similarities between the 12 context determinants and two reviews in the evolving literature (1, 21) influencing our decision to include all 12 determinants in the *SITS framework* (see Table 5). First, by comparison, two current reviews in the literature use similar definitions and or categorization for the 12 context determinants as those previously identified in the synthesis of eight F/M/Ts in the systematic review (hereafter referenced Nadalin Penno et al.) (7). Specifically, Squires et al. (21) uses the term ‘Domains’ and Shelton et al. (1) uses the term ‘Factors (themes)’, identifying similar determinants within the same categories/groupings, having similar definitions. This confirms the addition of the 12 determinants to similar constructs identified in the Nadalin Penno et al. (7) review incorporated into the *SITS framework*.

**Table 5:** Twelve sustainability determinants mapped to current reviews (1, 21).

**12 Sustainability Determinants** (Case Study Determinants mapped to Systematic Review Constructs identified in Nadalin Penno et al. (7)	Concept Analysis of “Context” (Squires, Graham et al. 2019) (21)	Emerging Sustainability Factors (themes) (Shelton et al. 2018) (1)
**Adopter Construct Determinants**: • student turnover (medical) • expert consultants • individual awareness/familiarity with innovation·population characteristics/needs/acuity level	**Domain = Providers within the Context** **Attribute** = People, **Feature** = Staffing composition **Attribute** = People **Feature** = Staffing qualifications & expertise **Attribute** = People **Feature** = Staffing qualifications & expertise **Domain = User of Context** **Attribute** = Patient Population, **Feature** = Patient/client demographics	**Implementor & Population Characteristics Factors** - Provider/implementor characteristics - Implementation expertise - Implementer characteristics - Population characteristics
**Leadership & Management Construct Determinants**: • leadership commitment (dept level);	**Domain = Internal Arrangement of Context** **Attribute** = Leadership, **Feature** = Active and Formal leadership	**Inner Context Factors** - Leadership/support
**Inner Context Construct Determinants**: • physical layout • competing internal priorities • team culture embraces innovation	**Domain = Internal Infrastructures/Networks** **Attribute** = Physical Infrastructure, **Feature** = physical structure **Attribute** = Social Infrastructure, **Feature** = formal organizational priorities **Attribut**e = Communications & Relationships, **Feature =** Social influence	**Inner Context Factors** - Structural Characteristic - Climate/culture - Climate/culture
**Inner Processes Construct Determinants**: • workload/staffing patterns • documented communication/ reporting systems;	**Domain = Internal Infrastructure/Networks** **Attribute** = Social Infrastructure, **Feature** = organization of care processes **Attribut**e = Communications & Relationships, **Feature** = formal communication	**Processes Factors** - Team Functioning - Communication
**Outer Context Construct Determinants**: • external pressure/demand from professional/regulatory bodies • goal alignment with external agencies.	**Domain = Broader System related to Context** **Attribute** = Market, **Feature** = competitive pressure **Attribut**e = Collaborative Relationship, **Feature** = collaborative practice	**Outer Factors** - Policy and legislation - Values, priorities, needs

Specifically, the ‘*Adopters*’ construct identified by Nadalin Penno et al. (7) continues to be uniquely categorized and defined as ‘users of the innovation’, which includes both providers and the consumers in the context in both published reviews (1, 21). For example, *Adopter* constructs comparisons in these two published reviews include: the “Domain: Providers or Users within the Context” (21), and the “Implementors and Population Characteristics Factors” (1). Second, ‘*Leadership*’ commitment or support for the innovation is also grouped separately by both reviews in the literature, either as an attribute within the “Inner Context” (1) or within the “Domain: Internal Arrangement of Context” (21). This finding further corroborates the previous distinction of *Leadership* as a separate context construct noted in the Nadalin Penno et al. (7) review, not evident in a previous concept analysis on healthcare innovation sustainability (23). Third, in the Nadalin Penno et al. (7) review the ‘*Inner Context’* construct includes internal structural determinants, separate from a ‘*Inner Processes*’ construct which includes established system or network determinants that exist to support the innovation. Similar determinant groupings for these two constructs are evident in both published reviews (1, 21). Lastly, a similar ‘*Outer Context*’ construct is evident across all three reviews (1, 7, 21). Alignment of these 12 context determinants with previous identified determinants (i.e., factors), definitions, and their categorizations in the current literature reviews (1, 21) reinforces their importance for sustainability. It further supports their addition to the 37 determinants identified in the Nadalin Penno et al. (7) review, resulting in a total of 49 (37 + 12) unique sustainability determinants presented in the *SITS* framework (see Table 4, and Figure 1).

### KTIs influencing sustainability in tertiary settings

#### Comparing 29 unique KTIs with the literature

Comparing the 29 KTIs to the ‘themes and approaches’ (constructs) identified in a review on the sustainability of approaches in healthcare by Lennox et al. (16) confirmed their inclusion in the *SITS framework*. The aim of the Lennox review was to identify studies that described approaches or strategies used related to sustainability in healthcare, and to describe the different perspective, applications and constructs within the approaches to guide future use by healthcare teams and researchers. The Lennox review included a total sixty-two publications each identifying a sustainability approach (e.g., 32 frameworks, 16 models, 8 tools, 4 strategies, 1 checklist, 1 process). The search included publications between 1989 and Sept 2017, having similar end dates in the systematic review (e.g., July 2018) (7). The majority of approaches (i.e., 37% or 23/62) were designed for use in general healthcare and did not specify a specific healthcare setting for use. Additionally, 31% (or 19/62) of the approaches were designed for use in public health settings, followed by 26% (or 16/62) of approaches designed for use in community settings. Only 3% (2/62) of the approaches were designed for use in acute care. Constructs across approaches were compared and 40 unique constructs for sustainability were identified. Comparisons across approaches (62) revealed 6 constructs that were included in over 75% (47/62) of the approaches regardless of the proposed interventions, setting or level of application. From their findings, Lennox et al. (16) developed a framework entitled, the “Consolidated Framework for Sustainability Constructs in Healthcare” (hereafter Lennox CF), which includes 6 themes and 40 constructs for sustainability. Thus, we compared the KTIs identified in the case study (15) to the 6 themes and 40 constructs identified in the Lennox et al. (16) review. Given the Lennox review (16) is the first review reported in the current literature identifying approaches for the sustainability of innovations in healthcare, we conducted a critical appraisal using the AMSTAR 2 rating tool (24). We determined a moderate to high confidence rating for the results (see Supplementary Material file S1).

We present four key considerations influencing the decision to include all 29 KTIs in the *SITS framework*. Details of the comparison of the 29 KTIs with the forty constructs reported in the Lennox CF (16) are presented on Table 6. First, the six themes identified in the Lennox CF (16) aligned with six constructs identified in the Nadalin Penno et al. (7) review, with minimal regrouping of the Lennox CF themes. This alignment confirms the applicability and relevance of the six constructs identified in the Nadalin Penno et al. (7) review to map these 29 KTIs to. Second, all 29 KTIs mapped to 17 (out of 40 constructs) constructs identified in the Lennox CF, that were evident in no less than 52%(32 out of 62) and as high as 90% (56 out of 62) approaches included in the Lennox et al. (16) review. Given the studies included in the Lennox review involved a range of settings, a variety of EBPs, and different levels of application, this alignment suggests potential relevance for the 29 KTIs beyond tertiary settings in other contexts, with other innovations, and level of application. Third, the 29 KTIs designed for use by acute care nurses in the case study (15) were not exact matches but rather considered similar in nature and several were grouped under the same construct. For example, 7 (of the 29) KTIs that included some form of ongoing training (e.g., eLearn modules, 1 on 1 training etc.) aligned with the Lennox CF construct entitled ‘Training and Capacity Building’. Fourth, only 2 out 62 studies (3%) included in the Lennox et al. (16) review were designed for acute care. Thus, the 29 KTIs identified in the *SITS framework* provide further specificity of KTIs designed for use in tertiary contexts, not evident in the Lennox et al. (16) review. This finding also highlights the need and importance of empirical research to further explicate the specific KTIs for sustainability in tertiary settings for acute clinical practice. Overall, the 29 KTIs included in the *SITS framework* provide further evidence to guide or inform future sustainability approaches and research for acute care.

**Table 6:** Integrated KTIs (*N* = 29) compared to Lennox et al, 2018 (16).

Systematic Review 7 constructs	Implementation Phase (0–2 years.) Department Level KTIs: Department RNs	Sustainability Phase (>2–10 years.) Department level KTIs: Department RNs	Sustainability Phase (at 10 years.) Unit level KTIs: Unit RNs	Lennox et al. 2018 Approaches for Sustainability (% = no. of studies using approach/total studies in review)	Lennox et al. 2018 6 Themes
	8 Imp/Sust KTIs + 4 Imp KTIs unique to Department RNs (*n* = 12)	8 Imp/Sust KTIs + 14 Sust KTIs unique to Department RNs (*n* = 22)	8 Imp/Sust KTIs + 3 Sust KTIs unique to Unit RNs (*n* = 11)		
**Innovation**	**Embedding of Pain P/P** **	**Embed ongoing refinements** **	** e**mbed prompts**	**• Intervention adaptation and receptivity 73% (45/62)**	Initiative Design
		** **	**Digitalized Pain P/P and forms**	**Integration with existing programs and policies 79% (49/62)**	
	**Interdisciplinary Pain P/P established**			Integration with existing programs and policies 79% (49/62)	
	**Use frameworks to ID barriers** to integrate into routine practices			Integration with existing programs and policies 79% (49/62)	
**Adopters**	**Secure internal financial** commitment—time and Human resources to			**Staff involvement 42%** $\begin{array}{c} Resource\;Staff\;26\text{\%}\;\\ Resource\;Time\;6\text{\%}\; \end{array}]\;74\text{\%}(46/62)$	The People Involved
			**Mentorship by senior nurses**	**• Relationships and collaboration and networks 65% (40/62)**	
	**Joint collaboration from all levels of nursing plus other disciplines** to develop departmental implementation plan**		**Engages IP stakeholder involvement** on cttees**	**• Stakeholder participation 79% (49/62)**	
**Leadership & Management**	**Formalize BPG Coordinator role** to**	NPP dept leaders **comparing survey results among units** created a sense of competition among unit leaders and users to improve unit**	**Leadership strategies** - Clinical Coordinator—dept level: - Clinical Care Leaders—unit level - Unit Managers—unit level**	**• Leadership and champions 73% (45/62)**	
**Inner Context**		**Performance Evaluation indicators** for monitoring		• **Accountability of roles and responsibilities 56% (35/62)**	The Organizational Setting
		**Unit leaders lead** dept and unit level **patient centered initiatives for pain care**		• Defining aims and shared vision 53% (33/62)	
	**Obtaining buy-in and Formalize nurse leaders’ involvement** on Steering Cttee**	**Corporate level Internal cttees’ support ongoing review of clinical tactics** support sustained use**	**Fostering an IP and EBP culture** among IP team to support Pain P/P**	**• Organizational values and culture 71% (44/62)**	
		**Dept determine EBP priorities**		**• Defining aims and shared vision 53% (33/62)**	
	Secure external funds** a) RNAO PBSO—secure operating funds for initial training and resource s to build capacity b) secure capital external financial support—for point of care surveying system	**Development of an electronic monitoring system** to measure nursing sensitive indicators provide monitoring of BPG adherence**		• **Funding 68% (42/62)** • **General resources 90% (56/62)**	The Resources
**Inner Processes**	** Pain Council established—Interdisciplinary taskforce** **	**NPP reps develop formal and informal education** initiatives at dept & unit level in 2014 performed by Pain Council.**	** Ongoing Education** to support Pain P/P use by NPP and Educators:**	**• Training and capacity building 76% (47/62)**	Negotiating Initiative processes and Initiative Delivery
	** Training Champions** **	**Trains 170 Unit level expertise** = Champions, educators, APNs, work across units**	**Ongoing Training** to support Pain P/P use by NPP and Educators:**	• Training and capacity building 76% (47/62)	
		*Ongoing pain care education support at dept and unit levels becomes tailored overtime i.e. 1 on 1, case studies		• Training and capacity building 76% (47/62)	
	** **	*Mandatory **eLearn training** system		• Training and capacity building 76% (47/62)	
	** **	*Unit specific training of staff provided **based on audit remedial action plans** to improve			
	** **	Develop unit specific **additional resources/tools** overtime		• General resources 90% (56/62)	
	Use **multi-modal approach to disseminate**			• Training and capacity building 76% (47/62)	
	** **	**Spread EBP** to additional areas		• Training and capacity building 76% (47/62)	
	**Established Pain BPG taskforce/workgroup** in NPP dept –**	** NPP and Unit Leaders facilitate/lead remedial action plan for under performing units** **	**Monitoring and evaluation:** Dept level - ongoing training to do survey Unit level - audit and feedback Unit level - Patient satisfaction survey results shared**	▪ **Monitoring progress overtime 84% (52/62)**	
	** **	Ongoing biannual staff **training to conduct prevalence survey**		▪ Monitoring progress overtime 84% (52/62)	
	** **	**NPP Establishes regular performance monitoring:**		▪ Monitoring progress overtime 84% (52/62)	
	** **	Timely reporting of prevalence survey results led to **course correcting changes**		▪ Monitoring progress overtime 84% (52/62)	
			**Establishing effective communications between providers**,	• Relationships and collaboration and networks 65% (40/62)	
**Outer Context**		**New evidence released—**integrate into BPG		**• Evidence base for the initiative 52% (32/62)**	The External Environment
		**Staff participation on a regional network**		**• Community participation 56% (35/62)**	
		**Benchmarking** to external sources best practices		• Evidence base for the initiative 52% (32/62)	
**Outcomes**					

** **8** Common KTIs across Implementation (Imp) (0–2 years.) and Sustained use phases (Sust) (>2–10 years. and at 10 years.)

## Discussion

It is apparent from this research that determinants and KTIs both influence the way in which healthcare innovations are sustained over time in tertiary settings. What really matters is how and what individuals within the departments and units do that impacts sustainability. It is important to understand the influences underlying the determinants in real world settings and how the focus of the KTIs must adapt and or evolve with the integration of an innovation at different levels of application (e.g., departmental verses unit level use), and over time. With this in mind, the *SITS framework* uniquely pairs or maps sustainability determinants with sustainability-orientated KTIs demonstrating how the focus varies with level of application (e.g., departmental use—across several units at one time, to unit specific level use) and over time (i.e., during implementation and sustained use phases) (see Table 4 and Figure 1). To our knowledge, the *SITS framework* provides the first theory and evidence informed comprehensive list pairing together sustainability determinants and related sustainability-orientated KTIs to guide practitioners and researchers sustain the use of EBPs in tertiary settings over time.

### Main observations related to 49 unique sustainability determinants

Seven main observations related to the 49 sustainability determinants influencing sustainability of EBPs in tertiary settings over time within the *SITS framework* include:

(i) Impact of context determinants on sustainment
(ii) Influence of three determinants and constructs over time;
(iii) Similarities among theoretical and empirically derived determinants
(iv) Sustainability and level of application (e.g., department and unit levels)
(v) Potential utility of the twelve determinants beyond tertiary settings
(vi) The influence of academic institutes on sustainability of EBPs
(vii) Collaboration with experts affects sustainability of EBPs

#### Impact of context determinants on sustainment

Adding the twelve determinants identified in the case study (15) to the 37 in the Nadalin-Penno et al. (7) review, previously derived from eight F/M/Ts related to sustainability of EBPs within acute care contexts, provides further conceptual clarity to the concept and the determinants influencing sustainability, suggested by researchers (1, 14). It also illuminates the importance of considering aspects of ‘local context’ that promote or inhibit the sustainability of EBPs in healthcare contexts to achieve desired program goals and population outcomes over time, recently purported by researchers (1, 14, 25). For example, the *SITS framework* demonstrates 78% (25 out of 32) of determinants influencing sustainability in tertiary settings lie within four ‘context’ constructs; namely *Adopters, Leadership &Management, Inner Context, and Inner Processes*. Determinants within these constructs varied among case study participants (15) providing insight into ‘why’ the sustained use of EBPs varied among department and unit nurses (subcases) within the same organization. Similarly, in a recent study by Shrubsole et al. (26), local internal context and individual (or adopter) determinants were identified as key factors influencing sustained use of an EBP among clinicians working within four different hospitals. These findings highlight the need to focus on the specific unit-level ‘context’ determinants influencing practice use (or not) before developing or choosing KTIs meant to integrate the EBP recommendations into routine practice, suggested by Lennox (16). Overall, the *SITS framework* further clarifies for practitioners and researchers what internal and external contextual determinants potentially influence the sustainability of healthcare EBPs in real-world tertiary settings, such as hospitals. In summary, understanding context does matter for sustainability of EBPs in acute clinical practice within tertiary settings!

#### Influence of three determinants and constructs over time

Three determinants identified in the case study (15) during the implementation use phase (0–2 years.) were identified as having an influence during the sustained use phases (i.e., >2–10 years., at 10 years.). They include: *need* for the innovation; *leadership commitment*; and *external demand or pressure* for the innovation. These three determinants are also evident in the Nadalin Penno et al. (7) review. This finding demonstrates the potential impact of these determinants during both the implementation and sustained use phases of an innovation in tertiary settings, suggested in the literature (7, 10, 27). Furthermore, the three determinants span three different constructs: the *Innovation*, *Leadership & Management*, and *Outer Context* respectively. Case study (15) findings revealed how KTIs efforts were adapted over time to improve adherence to the innovation (e.g., Pain BPG recommendations) with their level of application (e.g., department verses unit) triggered by the focus of the adopters/users. Thus, researchers and practitioners should be mindful of how the underlying constructs change or evolve over time and the impact on these three determinants for two reasons: (1) to gain a better understanding of determinants that may potentially influence healthcare innovation sustainability during both the implementation and sustained use phases, and (2) to inform how to best tailor KTI efforts for sustainability previously suggested in the literature (2, 17).

#### Similarities among theoretical and empirically derived determinants

Comparing determinants between the data sources revealed 68% (11 out of 16) alignment between those determinants identified as ‘common’; occurring in more than 4 F/M/Ts in the Nadalin Penno et al. (7) review, and those identified in the case study (15). This finding demonstrates that not all theoretically nor empirically derived determinants occur in similar settings. There is a need for further empirical investigation of the barriers and facilitators influencing sustainability within tertiary settings to refine the *SITS framework*. This finding demonstrates the importance of empirical research to build comprehensive theorical frameworks to guide practitioners and researchers in clinical practice, suggested by other researchers (4, 5, 10) and sustainability framework authors (7).

#### Sustainability determinants and level of application

The *SITS framework* contains sustainability determinants derived from both departmental and unit level nurses (i.e., level of application), a perspective not made explicit among known theoretical conceptualizations for sustainability.

##### Similarities

Two determinants reported among case study department and unit level nurses highlight the importance of ‘building capacity for an innovation through (i) *stakeholder motivation and commitment* to the innovation’, and (ii) ‘*leadership engagement at all levels*’ within the organization to promote sustainability over time (15). These empirical findings align with those identified in the systematic review (7), wherein the majority of F/M/Ts (5 or more) identified adopters (or individuals, stakeholders) *belief in* and *commitment towards* the innovation, and *leadership and management commitment at all levels* (e.g., Board, department, and unit level) as key determinants influencing sustainability. Furthermore, facilitating determinants, such as the positional influence of leaders who impart the value of the change to decision makers, and the network of support and or commitment provided by a range of stakeholders, reportedly influenced whether an innovation was sustained in practice in previous studies (28, 29). Case study findings also reinforced the shared commitment of all stakeholders, including leaders', across the organization to prioritize the innovation (e.g., EB care) contributed to a sustainability-promoting culture of shared accountability, also evident in previous studies (19, 29–34).

##### Differences

Differences identified by case study participants (15) reflected a viewpoint based on their respective roles and responsibilities related to the innovation. For example, determinants identified by department level nurses focused mainly on organizational-wide (*Inner Context*) and *Outer Context* influences, while determinants identified by unit nurses revealed their focus on the use of the *Innovation* at the clinical practice level with *Adopters*, within the *Inner Context*, and related *Inner Process* influences.

Specifically, department level or organizational-wide influences impacting sustainability of EPBs over time included: (i) *internal competing priorities* such as infection control rates*,* (ii) higher-level human resource concerns related to the *complement of nursing staff on units*, and (iii) the *frequent turnover of medical students* (e.g., clinical placement rotation changes). The following ‘Outer Context’ determinants affected sustainability over time: (iv) *goal alignment* for the innovation with education partners, (v) maintaining *connections with related networks*, (vi) *external pressure or demand* from accrediting, government and regulatory bodies, (vii) *external support or recognition* for their efforts from external stakeholders (e.g., Registered Nurses of Ontario)(RNAO) (15), and (viii) *compatibility of the innovation to meet consumer demand*. These departmental determinants reveal an ‘outward focus’ and insight into organizational-wide roles and responsibilities that positions department level nurse leaders “to act as conduits, linking outer and inner contextual influences” to ensure sustainability of the innovation over time in an ever-changing acute healthcare environment. Notably, leadership is identified in a previous study wherein the mid-level management role is described as being critical to enacting a tie between the unit level leaders and point of care (29). This finding highlights the importance of a separate construct for ‘*Leadership and Management*’ in the *SITS framework* for sustainment within tertiary contexts.

The nineteen sustainability determinants identified by unit nurses in the case study (15) instead, reflected an individual and internal perspective, focused mainly on the ‘innovation’ and nurses’ use of it within their unit. In essence, these determinants illuminate nurses' daily clinical practice' viewpoint. These nineteen determinants aligned with the *Innovation, Adopter, Inner Context, Inner Process* constructs in the *SITS framework* (see Table 4 and Figure 1).

***Innovation Determinants***: First and foremost, case study unit level nurses reported *perceived innovation benefit* to patients/family and or staff was important for sustainability of the EBP (15). This ‘*Innovation*’ determinant was identified in 5 F/M/Ts in the systematic review (7), and aligns with a recent study where hospital unit level hospital-based nurses previously reported continued benefits as an essential innovation characteristic for sustainability of BPGs (35).

***Adopter Determinants****.* Four out of the seven ‘*Adopter*’ determinants identified by unit nurses, aligned with sustainability determinants identified in the systematic review (7). They include (i) *stakeholder commitment* towards the innovation, (ii) *individual commitment* to the innovation*, (iii) individual competency* to perform the innovation, and (iv) the *internal cohesion* between individuals leads to increased performance. The following three out of the seven ‘*Adopter*’ determinants added to those previously identified in the systematic review (7): (v) *population characteristics* related to the use of the innovation, (vi) *user awareness and or familiarity* with the innovation, and (vii) the presence of *expert consultants.* Unit nurses reported *patient* (population) *characteristics*, such as their preferences or acuity level, influenced their use of the EBP (15). Patient involvement was identified in the recent review by Lennox et al. (16) in 16% (10 out of 62) of studies to influence sustained use of EBPs in clinical practice. A recent concept analysis on context related to research utilization in practice identified *expertise of providers* within the context as a key feature (21). In a recent review by Cowie et al. (14) that identified barriers and facilitators influencing sustainability of hospital based interventions, having the appropriate expertise and knowledge in order to deliver the innovation was identified in 44% (14 out of 32) of studies, and engaging all persons with innovation expertise was identified as a major facilitating factor underpinning sustainability in 47% (15 out of 32) of studies. Unit nurses also reported that education initiatives (e.g., mandatory eLearn modules, general hospital orientation, annual pain education days) offered to them supported the training of new nurses and updated *nurses' awareness* of policy refinements. These findings substantiate the importance of having an infrastructure that supports *user awareness and or familiarity* to perform the innovation suggested in the literature (2, 14, 36).

Additionally, in the case study unit nurses either reported the *internal cohesion between individuals* [e.g., senior nurse mentors, interprofessional team (IP) members], or *stakeholders' commitment* (e.g., formal clinical leader) facilitated their daily use of the Pain BPG recommendations (15). This finding reflects the unique difference observed regarding leadership support between the units. However, whether there is formal (managers) or informal (mentors and interprofessional team members) leadership support at the unit level, it is important to recognize the linkages and interactions between and attributes of these key individuals (e.g., managers, mentors) are important for sustainability among unit level nurses in tertiary settings. This highlights that EBP sustainability in nursing practice is often dependent on linkages between the persons (*Adopters*) and clinical processes and practices within the network of care it is situated in which has been identified in a previous study (35).

***Inner Context Determinants.*** Unit nurses indicated seven ‘*Inner Context*’ determinants influenced their use of the EBP. Five out of seven align with determinants identified in the systematic review (7). They included: having infrastructure supports for the innovations such as (i) *policies,* (ii) *equipment and supplies* (e.g., pumps), (iii) *shared cultural beliefs and or perceptions* towards the innovation (e.g., EB care), (iv) a *climate* that facilitated the EB care, and (v) a *culture that integrates the innovation into context norms* (documents, protocols, manuals). The remaining two ‘*Inner Context*’ determinants add to those identified in the systematic review (7): (vi) *the physical layout* of unit - between two floors, and (vii) having *a team culture* that embraced the innovation. These ‘*Inner Context*’ determinants further demonstrate that infrastructure supports and promoting a culture that embraces the innovation are needed to for successful sustainability of EBPs in clinical practice, reported by Lennox et al. (16), Shelton et al. (1), and Squires et al. (21).

***Inner Process Determinants.*** Unit nurses indicated four ‘*Inner Process*’ determinants influenced their sustained use of the EBP (15). Two that align with determinants in the systematic review (7) include: (i) having a *plan, method and schedule* to integrate the innovation and any updates or revisions into routine practices, and (ii) having *established communication system* to provide audit and feedback on adherence rates to EBP recommendations, and reporting processes for remedial plans. The remaining two ‘*Inner Process*’ determinants added to those in the systematic review (7): (iii) establishing *formal communication or reporting systems* to share innovation related patient information between practitioners (e.g., verbal shift reports) and between patients (e.g., in room care boards), and *(iv) workload or staffing patterns.* ‘*Inner Process’* determinants consisted of both formal (e.g., prevalence survey) and informal (e.g., verbal reports, care boards) systems. Establishing a means to monitor the long-term progress of the hospital-based innovations was identified in 59%(19 out of 32) of studies as one of the most frequently reported facilitating determinant for the sustainability of hospital-based innovations over time (14). Similar consistent reinforcement and feedback on maintaining EBPs provided to unit nurses by clinical leaders contributed to a sustainability-promoting culture of hospital-based innovations in other studies (29, 35).

#### Potential utility of the twelve determinants beyond tertiary settings

In the Squires et al. (21) review and concept analysis of context, they set out to examine the domains, attributes and features of context influencing research use (i.e., EBPs) among healthcare professionals. Seventy publications were included in the review and sources included several theories, models, tools, and studies from a variety of healthcare settings and countries, including a variety of EBPs, and different levels of application. A “Framework for Context” was developed comprised of 6 domains, 21 attributes and 89 unique features of the attributes, irrespective of setting, type of clinical EBPs, or professional roles (e.g., nurse, other healthcare team members) supporting a broader utility (21). Similarly, factors identified in the Shelton et al. (1) review included those from multiple settings and contexts, informed by the current evidence base (1). The twelve determinants reported by nurses in the case study (15) are similar to those identified in the two current reviews, potentially extending the utility of the twelve sustainability determinants in the *SITS framework* to other settings (1, 21), healthcare team members and EBPs (21).

#### Influence of academic institutions on innovation sustained use

The following observation is based on two (out of the twelve) determinants reported by nurses in the case study that influenced their use of the EBP in clinical practice: (i) *medical student turnover,* and (ii) *shared vision or goal alignment* (15). Partnerships are often established between healthcare agencies and educational institutions based on shared goals (e.g., provide EB care) and to facilitate medical student clinical placements, internships or residencies. It is not uncommon to expect medical trainees to implement EBPs. Case study nurses also reported frequent *medical resident team rotation changes* inhibited the sustained use of the EBPs on their units (15). As a result, EBP training offered during general hospital orientation and to students (all types) was required. This included completing mandatory eLearn modules to ensure congruence with the established Pain protocol or policy. These two context determinants are also identified in a current review (21) to influence the use of EBPs in clinical practice, reinforcing their importance for sustainment in complex ever-changing in acute care environments.

#### Collaboration with experts affects sustainability

Case study nurses reported having access to available ‘*expert consultants*’ on their unit supported their ongoing use of EBPs ten years post-implementation (15). With increasing complexity and acuity of acute inpatients care, management of patient outcomes often requires collaboration and interdependence of various disciplines, such as nurse champions, physicians, and specialty services such as acute pain service (APS) team. Over ten years, case study findings revealed 170 BPG nurse champions were educated and trained to provide unit level expertise on guideline use to unit team members (15). They also formalized two advanced pain management teams: acute and palliative care services, which physicians and nurses could access when needed, to support advanced pain management needs (15). Expert consultants is identified as an attribute in the two recent reviews either as “staff expertise” (21) or “implementor expertise” (1) and is evident in previous studies (9, 35, 37, 38). Others have also observed that engaging *supportive multiple stakeholders* in clinical processes with ‘identified roles’ such as *experts*, promotes ongoing use of healthcare innovations in clinical practice (16). Having expert consultants at the unit level reinforces the conclusion noted in previous studies, that nurses work is part of a larger network of interprofessional collaborative care, including experts, that ultimately can affect sustainability of EBPs (35, 39). Thus, this determinant provides further evidence collaboration among experts and other practitioners is often necessary to promote sustainabiltiy of EBPs in tertiary settings.

### Main observations related to 29 sustainability-orientated KTIs

We present seven main observations related to the 29 KTIs included in the *SITS framework* that effectively fostered change behaviors and facilitated sustainability of an EBP in tertiary setting over time. They include:

(i) Eight KTIs had continuous impact on sustainability;
(ii) Providing timely reporting and feedback promoted sustained use;
(iii) Using an incremental approach to address adherence
(iv) Using a user participatory approach influenced adherence;
(v) Monitoring adherence promoted accountability and built capacity for EB care;
(vi) Creating leadership accountability for EBP outcomes;
(vii) Unit informal practices or processes may unknowingly influence adherence measurement.

#### Eight KTIs had continuous impact on sustainability

In the case study, eight (out of 29) KTIs had a continuous impact during the implementation use phase (0–2 years) and sustained use phases (e.g., >2–10 years., at 10 years post implementation (15).These eight KTIs provide insight into how the focus of the KTIs evolved over time with the change in level of application (e.g., department-across units verses unit specific use) to fit within the context. This novel finding is important to consider when designing KTIs to be used in an ever-changing healthcare setting such as a hospital. To this end, the linking or tailoring of KTIs to promote, address, or overcome the identified determinants aimed at sustaining EBPs, such as BPGs, during the dynamic ongoing sustainability phase is a necessary step. The added value or effectiveness of tailoring KTIs over time to support the integration of the innovation into routine practices or processes (local context), previously identified as an implementation strategy to overcome barriers to change (40, 41), now adds to sustainability knowledge. Notably, the eight multi-layered KTIs used by departmental and unit level participants in the case study (15) to integrate the EBP into routine practices and over time facilitated sustainability. This finding exemplifies how the agents/actors, strategies, and changing contexts are interrelated suggested by Mielke et al. (25) in a recent study examining the successful and sustainable implementation of complex innovations or interventions in dynamic contexts. Findings also add credence to the conceptualization that sustainability of healthcare innovations in clinical practice is as an “*ongoing dynamic process*” suggested in the systematic review (7), evident in existing sustainability frameworks (19, 23, 33, 42, 43), and the literature (14, 25, 44).

#### Providing timely reporting and feedback

The *timely reporting and feedback* of performance data (e.g., prevalence survey, patient satisfaction results) to clinical leaders and unit nurses and comparing of results among units created ‘a sense of competition’ that spurred a chain of activities to improve (15). Specifically, ongoing changes in measurement activities became more focused and sophisticated to target selected EBP recommendation behaviours*.* Additionally, establishing a point of care *monitoring system* that provided *regular reports on adherence rates* to EBP recommendations produced the necessary data critical to determine remedial action plans (a *feedback mechanism*) for the sustained use of the EBPs at the unit level (i.e., local context) (15). These KTIs are congruent with evidence in the literature pertaining to both phases. Specifically, studies have previously identified *audit and feedback strategies* (i.e., KTIs) effectively contribute to the uptake of EBPs during the implementation phase(Powell et al., 2015) and the sustained use phase (16) in clinical practice. Fleiszer et al. (35) also reports *regular feedback on BPG audit results* reinforced expectations and promoted sustained use of BPGs among nurses in a tertiary setting (hospital).

#### Using an incremental approach to address adherence

The *use of an incremental approach* to influence adherence to EBP recommendations shifted the focus and design of KTIs over time (15). For example, KTI efforts in the case study during implementation (0–2 years.) were focused on integrating recommendations into existing organizational-wide documentation and orientation processes and practices. However, during the sustained use phase, the linking of KTIs to targeted behaviors (i.e., focusing efforts on one recommendation at a time) at the department level over time (i.e., *an incremental approach*) while subsequently designing KTIs to address unit specific level low adherence rates (i.e., adapting KTIs to unit specific routines, practices, and processes) promoted sustainability (15). This change reflects the realization that it is impossible for an organization to obtain high adherence to all BPG recommendations, on all units, at the same time. The integration and adaptation of the innovation into existing organizational programs and policies (i.e., routine practices and processes) at the department and unit levels was identified as key KTIs or approaches in the Lennox et al. (16) review, in 79% and 73% of studies respectively, regardless of the innovation, or setting. The ongoing use of these eight KTI demonstrates how innovation integration and adaption is also necessary for sustainability of EBPs in tertiary settings, adding to the existing knowledge.

#### Use of a user participatory approach facilitates sustainability

The use of a *user participatory approach* to engage leaders and users in the development of KTIs to enhance adherence to EBPs facilitated sustainability in the case study (15). For example, at the department level, engaging users on EBP committees and or taskforces initially mandated to develop a multi-modal approach to disseminate EBPs, and later to monitor guideline adherence rates and related patient outcomes, reportedly promoted commitment to Pain BPG and its sustained use over time. At the unit level, the use of a participatory approach encouraged unit nurses and other team members to collectively develop and tailor KTIs (i.e., remedial plans) to address low adherence rates to selected target behaviors (15). Promoting a ‘*user participatory approach*’ as a means to promote guideline use, also evident in the literature (45, 46), seems to be an effective means for EBP sustainability beyond the implementation phase. These findings confirm the notion that to produce real-world change over time there is a “need to consider staff and system domains as active components in the change process rather than imposing change” (45) for sustainability.

#### Monitoring adherence promoted accountability and built capacity for EB care

Case study participants reported the combined training of nurses to be surveyors to conduct the biannual audits (e.g., monitoring) served to increase their accountability towards sustaining EBPs in clinical practice while building their capacity for EBP use within their setting (15). Fleiszer et al. (35) also reports using nurses as auditors served to strengthen accountability. Training is identified as a key KTI in sustainability of healthcare innovations by several researchers (14, 16, 19, 47, 48). In the Lennox et al. (16) review, monitoring progress using a standardized mechanism, such as a prevalence survey, was identified in 84% (52 out of 62) of approaches as a key strategy for the sustainability of innovations in healthcare. In a recent review by Lynn et al. (18), measuring EBP recommendations at multiple time points is necessary to adjust for the adaptation of the EBPs, changes within the local context, and determining continued benefits on patient outcomes over time. Thus, the combination of KTIs (e.g., training and monitoring) should be an important consideration for sustained use of EBPs among unit level nurses in changing tertiary settings.

#### Creating leadership accountability for EBP outcomes

The inclusion of an EBP-related performance criterion into the performance evaluation system of leaders, had a trickled down impact on frontline staff performance expectations, critical to the process of change, creating an institutional system that held leadership and users accountable (i.e., responsibility for one's actions and to answer to someone with more authority) for the sustained use of EBPs (15) at both levels (organizational and unit). This KTI focused on obtaining shared accountability (e.g., getting buy-in) among stakeholders to deliver the innovation (e.g., Pain BPG) in support of the organization's vision for EB care. The use of an EBP criterion for individual performance evaluation is not explicitly identified as a KTI in a recent review of sustainability approaches, rather the literature suggests “incentives” and or “job requirements” are necessary for sustainability of EBPs (16). Thus, the EBP performance criterion exemplifies how to design a KTI for use in tertiary settings to promote use of EBPs in clinical practice. This KTI is congruent with other studies wherein point of care nursing leaders promoted shared accountability by reinforcing the expectation of EB care as the practice standard on their units using multiple strategies, one of which included evaluating performance (29, 35).

#### Unit informal practices or processes may unknowingly influence adherence measurement

The assumption case study nurses were not carrying out EBP recommendations could not be drawn solely based on the low adherence rates derived from the audited results (15). In fact, reported unit level practices and processes related to EBP recommendations not recorded in the health record (e.g., use of clipboards, whiteboards, and verbal reports) provided insight into low adherence rates (15). The accuracy of nursing documentation among acute care nurses has been studied in similar acute care settings (49–51). Doran (51) and Paans (49, 50) have reported low rates or scores related to the accuracy of nursing intervention documentation. Doran et al. (51) further indicated that nurses' documented ‘assessments of patient status’ more frequently than the ‘nursing interventions they were preforming’. Examination at point of care is needed to determine whether low adherence rates are due in part to a lack of accurate documentation. If so, effective KTIs to enhance or formalize documentation are required. More recently, the literature suggests it is important to routinely monitor KTIs such as these that facilitate or inhibit sustainability of EBP in acute care contexts (14). This is an important consideration for healthcare innovation sustainability given similar informal processes and or practices are likely common in many similar healthcare settings and not part of the formal documentation system.

## Implications

### Nursing leadership and practice implications

The implementation and sustainability of EBPs is a complex process. It requires the continued commitment and efforts of multiple supportive stakeholders across the organization from Board to unit level individuals. Establishing and supporting structural processes (e.g., systems to monitor the innovation) and infrastructure supports (e.g., policies, procedures, human resources) seems necessary to build capacity and a culture of shared accountability for the outcomes of sustaining the use of EBPs across the organization. Using a participatory approach to engage users of EBPs to participate on related committees and taskforces to support ongoing review of clinical tactics also facilitates buy-in promoting sustainability. Providing ongoing education and training at the organizational-wide (e.g., orientation sessions, education days) and unit level (e.g., one on one training, in-services) are needed to build capacity as well. Establishing an audit and feedback system that uses an incremental approach to guide ongoing efforts to address low adherence over time should also be considered. Finally, establishing an institutional system that reinforces leadership's commitment to sustaining EBPs, such as the use of a performance criterion or a requirement to report the impact of the use of the EBPs on patient outcomes as part of the organization's quality reporting system, promotes healthcare innovation sustainability.

#### Clinical practice level considerations for sustainment

##### Unit leader considerations

To achieve sustained use of EBPs at the point of care it is important to realize sustainability is dependant on the unit's team-wide efforts, not just an individual unit nurse's adherence to guidelines. Sustaining EBPs can be maximized if unit leaders maintain a unit-wide perspective on how recommendations are being integrated into daily routines, processes and practices. Unit level leaders (e.g., managers, champions, educators) should adopt strategies that promote use of EBP recommendations in regular and responsive ways to support ongoing use. For example, utilizing daily interprofessional patient rounds to discuss EBP related clinical management issues. Additionally, given conditions underlying sustainability determinants change over time, leaders also need to focus on establishing strategies that build capacity and accountability among Interprofessional (IP) team members to ensure sustained use. For example, establishing unit specific EBP priorities for monitoring, evaluation and collaborating with unit teams on developing remedial KTIs to address low adherence, and or to set benchmarks builds capacity. Encouraging unit nurses to participate in regular monitoring and evaluative processes (e.g., audits), on units not their own builds capacity and fosters accountability for EB care, promoting sustainability. Conclusively, unit leaders' efforts should focus on promoting a ‘culture of shared accountability’ for the ongoing use of EBPs among all team members to enhance sustainability at the practice level.

##### Unit nurse considerations

Unit nurses should be encouraged to participate in the establishment and ongoing revisions of EBP polices or protocols and determining the measurable indicators for each recommendation to be surveyed. Engaging unit nurses to identify established processes and practices related to EBP recommendations on their units and how to best to integrate *prompts* will promote sustained use. Attention to established informal practices and processes related to EBP recommendations that are not documented in the health record can provide insight into low adherence rates and provide a focus for how best to design KTIs that promote formal documentation of nurses' ongoing point of care related intervention efforts. Given increasing complexity, patient acuity levels, workloads, and time barriers in tertiary settings, it is imperative KTIs related to documenting recommendation efforts are flexible and motivational for nurses to carry out. Use of frameworks by unit nurses to identify barriers to guide sustainability efforts such as developing course correcting KTIs designed to incrementally address low adherence rates (e.g., tailoring of KTIs) facilitates sustained use. Encouraging unit nurses to participate in ongoing EBP education and training to become champions to provide expertise at the unit level is necessary to maintain awareness of refinements and new evidence at the unit level over time. Training unit nurses and IP team members to be surveyors to conduct the EBP prevalence audits promotes increased accountability towards sustaining EBPs in clinical practice while building their capacity for EB care within the setting.

Moreover, these ongoing internal efforts to improve patient outcomes that target collaboration among leaders, unit nurses, and IP team members for evidence-based care promotes sustained use of EBPs in acute clinical practice in tertiary settings. In short, sustainability depends on the linkages, shared actions, and social influence of teams among unit leaders at the department and unit level, along with the nurses and IP team members at the point of care.

### Strength and limitations

To our knowledge this is the first framework that pairs determinants, whether a facilitator or barrier to promote the sustained use of an EBP over time, to related KTIs for use in tertiary settings adding to the current knowledge. Sustainability determinants and related KTIs were derived from the synthesis and comprehensive analysis of healthcare sustainability F/M/Ts (7) and an in-depth, theory informed empirical study (15) which focused primarily on sustainability of an EBP in an acute care context. The resultant *SITS framework*, consists of seven sustainability constructs, forty-nine unique determinants, and twenty-nine unique KTIs primarily related to tertiary settings (see Figure 1). Novel insights are presented regarding the relationship between determinants, their level of application (i.e., organizational wide vs. unit level) and ‘how’ the focus of the related KTIs must evolve over time to resolve the fit between the EBP and the changing context during both phases. The eight KTIs identified that continuously impacted the sustainability of an EBP over time are important to consider when designing KTIs to be used in ever-changing healthcare settings. *The SITS framework* further confirms that healthcare innovation sustainability is an “ongoing phase” that occurs post the initial implementation use phase (beyond 0–2 years). Moreover, the *SITS framework* can be used as a practical guide or check list for those planning or currently implementing EBPs.

There are limitations to consider when using the *SITS framework*. First, the systematic review and theory analysis included sustainability F/M/Ts published by July 2018, and was restricted to four key databases, known to focus on healthcare and or implementation science. Thus, F/M/Ts from social science and management literature may have been missed. Second, the focus on one BPG, within one multi-site healthcare organization, from solely a nursing perspective is a limitation. However, unlike other BPGs, the Pain BPG was uniquely implemented across all inpatient units which we believe would have broad application to a variety of nursing environments, and results would serve to advance knowledge on the long-term sustainability of nursing BPGs. The applicability and refinement of the *SITS Framework* among other healthcare settings is recommended. Third, this research was not focused on differentiating the level of application related to findings, further clarification is needed. Instead, the design focused on having department and unit level nurses identify the unique sustainability constructs, determinants, and KTIs that effectively influenced sustained use of an EBP in their tertiary setting across all units over time and at the unit level at the ten year timeframe. Lastly, another limitation is the ‘Outcome’ construct remains underdeveloped in part due to the focus on a single practice guideline; the internal and external pressures unique to the Pain BPG; and the lack of evidence focused on this construct to date.

### Future directions for sustainability research

Sustainability is an evolving field of research within implemenatation science. Understanding and measuring how sustainability research efforts can enhance progress towards improved patient outcomes is critical. To advance sustainability knowledge future inquiry should focus on the following the following five directions. First, further investigation in multiple tertiary settings is required to provide additional empirical evidence, to refine the *SITS framework* constructs and determinants, to inform the pairing of determinants and related sustainability KTIs or approaches, and to confirm generalization. Second, one of the eight KTIs identified as having an impact on sustained use of an EBP over time (e.g., use of prompts in formal documentation) should be selected to inform the design of an intervention study to explore applicability and further framework refinement. Third, future research is needed to further clarify and differentiate how a similar KTI is used by the different level actors and their role at the different level of application (organizational verses unit) to refine the *SITS framework*. Fourth, to understand the impact of implementation on sustainability of healthcare innovations, an examination of F/M/Ts containing both implementation and sustainability constructs and determinants for tertiary settings should be undertaken using a similar theory analysis approach (52). Results could then be compared to the *SITS framework* and interpretations made regarding potential overlap and or impact of implementation on sustainability, and further substantiate insights revealed in the *SITS framework*. Fifth, to inform the Outcome construct in the *SITS framework*, further examination is recommended to explicitly identify related sustainability indicators, previously supported in the literature by framework authors (19, 23, 34, 42) and researchers (1, 7). Focus should be on determining the level of influence or impact of an EBP on specified outcomes or type of outcomes (e.g., service or patient outcomes) post implementation (e.g., >2 years.), at any one of the four levels of change (e.g., individual, team or department, organization-wide, or system level) identified by Proctor et al. (27). Much remains to be learnt about this complex concept of sustainability. More focus is needed to understand the dynamic interactions between and among determinants across a variety of contexts and to evaluate planned KTIs to support the sustainability of healthcare innovations in real-world settings over time.

## Conclusion

### How *SITS framework* contributes to current knowledge

The *SITS framework* consists of seven sustainability constructs, forty-nine unique determinants, and twenty-nine unique related KTIs necessary to sustain EBPs in tertiary settings. It provides further conceptual clarity, and corroborates the recommendation by researchers (7, 14) that sustainability is a dynamic process or phase to add to the current sustainability definition by Moore et al. (3). The *SITS framework*, as a novel resource, has practical implications for researchers, practitioners and administrators when designing, implementing and sustaining healthcare innovations, such as EBPs, for clinical practice in tertiary contexts. The majority of the forty-nine sustainability determinants identified are within the 5 ‘context’ constructs, providing insight into “*why*” the sustained use of EBPs may vary among units and departments within the same or different setting. It also highlights the need to focus on the specific unit level contextual determinants influencing use (or not) before developing or choosing KTIs or approaches to effectively embed an EBP into routine practice if one expects to sustain its use over time. Additionally, the three key determinants identified as having a continuous influence during both the implementation and sustained use phases: a *need* for an innovation (e.g., EBP), *leadership commitment*, and *external demand or pressure* for the innovation, are important considerations for sustained use of EBPs in tertiary settings. Moreover, practitioners and researchers not only need to be mindful of the relationship between or among determinants, but the underlying conditions influencing determinants within the constructs over time for sustainability of healthcare innovations to prevail.

More importantly, the *SITS framework* highlights sustainability of EBPs in clinical practice does not rest solely on identifying the determinants influencing use, but “*how*” one manages the determinants over time matters. Specifically, determinant identification is only part of the equation for healthcare innovation sustainability, developing effective KTIs to improve nursing practice and related patient outcomes is the other critical part. Linking and tailoring of KTIs to promote, address, or overcome the identified determinants aimed at sustaining EBPs during the dynamic ongoing sustainability phase is a necessary step. Twenty-nine KTIs promoted sustained use of the EBP in tertiary settings, eight KTIs had a continuous impact during implementation phase (0–2 years), the sustained use phases (>2–10 years, at 10 years). The eight KTIs provided insight into “*how*” the focus of the KTIs evolved over time with the change in level of application (e.g., across units or departmental verses unit specific application) to fit within the local context. This is important to consider when designing KTIs to be used in an ever-changing acute healthcare context.

Together determinants and KTIs, undoubtingly do influence the way in which healthcare innovations are sustained. It is important to understand the influences underlying the determinants in real world settings and how the focus of the KTIs must evolve with the integration of an innovation at different levels of application and over time. Given healthcare innovation sustainability is a ‘process’ or ‘ongoing stage’, what really matters is “how” and “what” the organization does to sustain the innovation at all levels over time within ever-changing tertiary settings.

## Data Availability

The original contributions presented in the study are included in the article/**Supplementary Material**, further inquiries can be directed to the corresponding author.
