# Supplementary material for: The SITS framework: sustaining innovations in tertiary settings
Source: Front Health Serv. 2023 Jun 8;3:1102428. doi: 10.3389/frhs.2023.1102428 (PMC10287174; doi:10.3389/frhs.2023.1102428)
Supplement: Supplementary file 1 [file Datasheet1.docx]

**Supplemental material file 1**. Critical appraisal of review (Lennox et al., 2018)

AMSTAR 2: a critical appraisal tool for systematic reviews that include randomised or nonrandomised studies of healthcare interventions, or both

Overall rating = Moderate to High in critical domains given item 15 is partially related

**Box 1 AMSTAR 2 critical domains**

- Protocol registered before commencement of the review (item 2) Yes
- Adequacy of the literature search (item 4) Yes
- Justification for excluding individual studies (item 7) Yes
- Risk of bias from individual studies being included in the review (item 9) No
- Appropriateness of meta-analytical methods (item 11) NA
- Consideration of risk of bias when interpreting the results of the review (item 13) Yes
- Assessment of presence and likely impact of publication bias (item 15) NA but Yes discussed Publication bias as only published studies included

**Box 2 Rating overall confidence in the results of the review**

- **High**
- *No or one non-critical weakness*: the systematic review provides an accurate and comprehensive summary of the results of the available studies that address the question of interest
- **Moderate**
- *More than one non-critical weakness**: the systematic review has more than one weakness but no critical flaws. It may provide an accurate summary of the results of the available studies that were included in the review
- **Low**
- *One critical flaw with or without non-critical weaknesses*: the review has a critical flaw and may not provide an accurate and comprehensive summary of the available studies that address the question of interest
- **Critically low**
- *More than one critical flaw with or without non-critical weaknesses*: the review has more than one critical flaw and should not be relied on to provide an accurate and comprehensive summary of the available studies
- *Multiple non-critical weaknesses may diminish confidence in the review and it may be appropriate to move the overall appraisal down from moderate to low confidence

Lennox, L., Maher, L., & Reed, J. (2018). Navigating the sustainability landscape: a systematic review of sustainability approaches in healthcare. *Implementation Science : IS*, *13*(1). <https://doi.org/10.1186/s13012-017-0707-4>
